# Tissue-engineered small-diameter vascular grafts containing novel copper-doped bioactive glass biomaterials to promote angiogenic activity and endothelial regeneration

**DOI:** 10.1016/j.mtbio.2023.100647

**Published:** 2023-04-27

**Authors:** Neda Alasvand, Aliasghar Behnamghader, Peiman B. Milan, Sara Simorgh, Ali Mobasheri, Masoud Mozafari

**Affiliations:** a Bioengineering Research Group, Department of Nanotechnology and Advanced Materials, Materials and Energy Research Center (MERC), Tehran, Iran; b Cellular and Molecular Research Center, Iran University of Medical Sciences, Tehran, Iran; c Department of Tissue Engineering & Regenerative Medicine, Faculty of Advanced Technologies in Medicine, Iran University of Medical Sciences, Tehran, Iran; d Research Unit of Health Sciences and Technology, Faculty of Medicine, University of Oulu, Oulu, Finland; e Department of Regenerative Medicine, State Research Institute Centre for Innovative Medicine, Vilnius, Lithuania; f Department of Joint Surgery, First Affiliated Hospital of Sun Yat-sen University, Guangzhou, China; g World Health Organization Collaborating Centre for Public Health Aspects of Musculoskeletal Health and Aging, Liege, Belgium

**Keywords:** Vascular grafts, Bioactive glass, 3D-printing, Electrospinning, Endothelium regeneration, Angiogenesis, Antibacterial

## Abstract

Small-diameter vascular grafts frequently fail because of obstruction and infection. Despite the wide range of commercially available vascular grafts, the anatomical uniqueness of defect sites demands patient-specific designs. This study aims to increase the success rate of implantation by fabricating bilayer vascular grafts containing bioactive glasses (BGs) and modifying their composition by removing hemostatic ions to make them blood-compatible and to enhance their antibacterial and angiogenesis properties. The porous vascular graft tubes were 3D printed using polycaprolactone, polyglycerol sebacate, and the modified BGs. The polycaprolactone sheath was then wrapped around the 3D-printed layer using the electrospinning technique to prevent blood leakage. The results demonstrated that the incorporation of modified BGs into the polymeric matrix not only improved the mechanical properties of the vascular graft but also significantly enhanced its antibacterial activity against both gram-negative and gram-positive strains. In addition, no hemolysis or platelet activity was detected after incorporating modified BGs into the vascular grafts. Copper-releasing vascular grafts significantly enhanced endothelial cell proliferation, motility, and VEGF secretion. Additionally, *In vivo* angiogenesis (CD31 immunofluorescent staining) and gene expression experiments showed that copper-releasing vascular grafts considerably promoted the formation of new blood vessels, low-grade inflammation (decreased expression of IL-1β and TNF-α), and high-level angiogenesis (increased expression of angiogenic growth factors including VEGF, PDGF-BB, and HEBGF). These observations indicate that the use of BGs with suitable compositional modifications in vascular grafts may promote the clinical success of patient-specific vascular prostheses by accelerating tissue regeneration without any coagulation problems.

## Highlights

• Modified BGs improve the mechanical properties and increase the antibacterial activity of the vascular grafts.
• Vascular grafts containing modified BGs enhance endothelial cell migration and angiogenic potential.
• Vascular grafts containing modified BGs greatly decrease the expression of inflammatory proteins (IL-1β and TNF-α).
• Vascular grafts containing modified BGs increase the expression of angiogenic factors in vascular grafts (VEGF, PDGF-BB, HEBGF).
• Vascular grafts containing modified BGs accelerate tissue regeneration in patient-specific vascular prostheses.

## Graphical abstract

**Figure:**
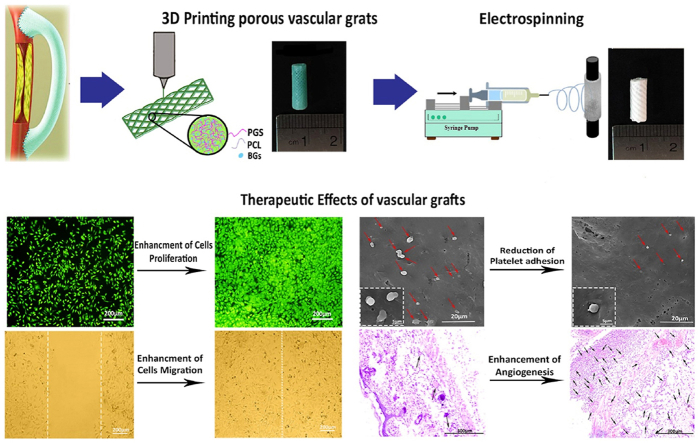

## 1 Introduction

Cardiovascular diseases have been considered one of the leading causes of death worldwide. In severe cases, bypass surgery is required to improve the constriction and hardening of the blood arteries [1]. Artery segments and autologous veins are yet the benchmarks for grafts in cardiovascular operations, despite the fact that these approaches are not always accessible due to previous harvests, disease conditions, or anatomical heterogeneity. Additionally, within the first 12–18 months after surgery, saphenous vein grafts’ rate of failure, which is the most commonly utilized vascular for coronary artery bypass, approaches 25%, demonstrating the critical need for new treatment methods [2]. Expanded polytetrafluoroethylene (GORE-TEX®), polyurethane, and poly (ethylene terephthalate) (Dacron) are amongst the synthetic vascular prostheses presently offered. Due to the infection, neointimal hyperplasia, slow re-endothelialization, and poor blood compatibility, remodeling and growth capacities of living tissues are absent in these vascular prostheses and they are not ideal as vascular grafts with small diameters (<6 ​mm) [3].

Biodegradable small-diameter vascular grafts were developed as a result of the pervasive and significant unmet demand for surgical intervention in severe vascular disease, especially when autologous transplants are not an achievable option. Fabrication of a flawless blood-compatible surface has proven to be challenging, and no off-the-shelf graft has yet surpassed the performance of an autologous vessel. Due to the fact that an endothelial layer is the largest barrier to blood clotting *in vivo*, numerous researchers have attempted to replicate the complete endothelium on synthetic vascular grafts [4]. Migration of endothelial cells (ECs) is a crucial component of vascular graft endothelialization and may occur via anastomotic cell infiltration, transmural microvascular ingrowth, circulating cell adhesion, or ex vivo cell seeding [5]. Preferably, the promotion of endogenous migration *in vivo* is preferred as it removes the need for ex vivo graft and cell treatments. Cell migration can occur via transmural capillary ingrowth (such as cell migration through the graft wall) or by anastomosis at the graft–vessel junction, according to the hypothesis [6]. As a consequence, numerous attempts have been made to enhance endothelialization by the administration of growth factors like vascular endothelial growth factor (VEGF) and fibroblast growth factor (FGF). Nonetheless, growth factors are expensive and the most effective delivery routes are uncertain [7]. It appears that bioactive glasses (BGs) containing particular ions, could provide reliable alternatives for expensive growth factors used in spontaneous endothelialization [8,9]. Numerous studies indicate that BGs can promote blood vessel development, which is essential for tissue regeneration and the healing of soft tissue lesions [10,11]. In comparison to silicate-based BGs, borate-based BGs have attracted more interest due to their marginal chemical stability and rapid degradation, resulting in the release of essential ions [12]. All of the ions present in borate-based BGs have a significant effect on biological processes, such as endothelialization and angiogenesis [5]. Boron (B) has many biological effects, including the acceleration of wound healing *in vivo*, the activation of growth factors and cytokines, an increase in RNA production, and the regeneration of extracellular matrix [11]. In particular, B is critical for ECs proliferation and migration, as well as increasing VEGF and transforming growth factor (TGF-1) [8]. Magnesium (Mg), another ion found in BGs, is required for cell cycle regulation in various cell types and promotes ECs proliferation. Mg has also been shown to be capable of preventing thrombosis in animal tests [13]. According to research, when Mg is exposed to ECs, it produces more nitric oxide (NO) [14,15]. During the first 6 ​h following implantation, the blood-contacting surface of implanted biomaterials is a competitive location for bacterial adherence and infection, as well as a site for host cell attachment and tissue integration. This competitive stage is important for implant integration with the surrounding tissue, as the formation of bacterial biofilm on implants prevents tissue integration and leads to implant failure [6]. As a result, a new issue in cardiovascular tissue engineering is developing biomaterials with a designed surface to enhance tissue integration while eliminating bacteria growth and infection. For this purpose, Copper (Cu) is a well-known antimicrobial material that can be applied against fungi and gram-negative and gram-positive bacteria [16,17]. Among the many factors involved in angiogenesis that Cu regulates are fibronectin, fibroblast growth factor (FGF)1/2, vascular endothelial growth factor (VEGF), prostaglandin E−1, collagenase, angiogenin, and ceruloplasmin; these are the ones primarily responsible for initiating the process (via vascular permeabilization and vasodilation), guiding its development (via mobility, ECs growth, and morphogenesis [18]. Despite the desirable advantages described above, BGs have not been previously used in vascular graft tissue engineering because of the presence of several ions in their structure that has hemostatic effects, which restricts their use in blood-contacting applications. These ions include silica (activates coagulation factor XII), phosphate (begins the extrinsic pathway), and calcium (activates the intrinsic system) [19]. Therefore, in order to employ BGs in blood-contacting purposes and avoid coagulation, it is necessary to synthesize them with modified composition, in which these ions must be substituted or removed.

Fabrication of vascular grafts has been done using a variety of approaches [20]. A precise design and production approach, for instance three-dimensional (3D) printing, is more likely to be preferred to handle more complicated vascular network architecture and its detailed parameters, like specific curvatures or bifurcations, and the unique defect anatomy of each patient. Currently, 3D printing has evolved into a low-cost, high-speed manufacturing process capable of producing patient-specific medical implants in small batches, while wasting fewer materials, implementing higher dexterity over spatial complexity, and patient-specific mechanical and biological characteristics. By using 3D computer-aided design (CAD) models, 3D printers use successive layer deposition to create high-resolution biodegradable materials [21]. So far, various biomaterials have been used to fabricate vascular grafts [22]. Among these biomaterials, the biodegradable and biocompatible blend of polycaprolactone (PCL) and polyglycerol sebacate (PGS) has received vast attention in cardiac tissue engineering [23]. This blend has mechanical properties that are comparable to those of cardiovascular tissue and is biodegradable and biocompatible [24]. Furthermore, research has demonstrated that PGS-PCL scaffolds promote the proliferation of human mesenchymal stem cells (hMSCs) [25] and human umbilical vein endothelial cells (HUVECs) [26]. As a result, the blend offers potential for vascular graft engineering.

The object of this research is to develop a strategy for fabricating a bilayer vascular graft with a 3D-printed porous layer comprising PGS, PCL, and modified BGs as the inner layer to accelerate the remodeling process. To ensure adequate blood sealing, the 3D-printed layer was wrapped with an electrospun PCL nanofiber sheath. The modified BGs in the 3D-printed layer of the vascular grafts could enhance the mechanical and biological properties of the vascular grafts, consequently improving clinical safety.

## 2 Materials and methods

### 2.1 Modified copper-doped BGs synthesis

Melt-quench was used to make the modified BGs from the 13–93B3 borate BG framework. According to our previous work, modified borate BGs were made using B_2_O_3_, MgO, K_2_O, Na_2_O, and CuO, while hemolytic components like calcium, phosphate, and silica were removed [27]. Sigma-Aldrich UK supplied reagent-grade precursors. Each group of modified BGs was carefully mixed before melting in an electric furnace at 1200 ​°C for 2 ​h. Modified BGs were quenched in water to make frits after melting. Planetary balls milled and sieved all frits. B0, B1, B2, and B3 are the different groups of the modified BGs, and their compositions are listed in Table 1.

**Table 1:** The compositions of the modified BGs and the list of vascular grafts abbreviations.

Modified BGs (wt%)	Vascular grafts	Abbreviation
**B0 (60B**_**2**_**O**_**3**_**, 16MgO, 18K**_**2**_**O, 6Na**_**2**_**O)**	PCL-PGS-B0	P:P:B0
**B1 (58.2B**_**2**_**O**_**3**_**, 15.5MgO, 17.5K**_**2**_**O, 5.7Na**_**2**_**O, 3CuO)**	PCL-PGS-B1	P:P:B1
**B2 (57.1B**_**2**_**O**_**3**_**, 15.2MgO, 17.2K**_**2**_**O, 5.5Na**_**2**_**O, 4.8CuO)**	PCL-PGS-B2	P:P:B2
**B3 (54.5B**_**2**_**O**_**3**_**, 14.5MgO, 16.7K**_**2**_**O, 5.1Na**_**2**_**O, 9 CuO)**	PCL-PGS-B3	P:P:B3
**-**	PCL-PGS	P:P

### 2.2 Fabrication of the **bilayer** vascular grafts

To prepare PGS, PCL, and the modified BGs ink, PGS was first synthesized according to the procedure that was previously reported [23]. In brief, an equimolar mixture of sebacic acid (Sigma Aldrich, purity 99%) and anhydrous glycerol (Sigma Aldrich, purity 99%) was stirred and heated to 120 ​°C in the presence of bubbling N_2_ for a day. The reaction mixture was stirred at 120 ​°C at 1 ​Torr for another day to form the feint yellow, highly viscous semisolid pre-polymer.

Then, each BG was dissolved in 10 ​ml of dichloromethane (Sigma Aldrich) and 50 ​μl of dimethyl sulfoxide (DMSO, Sigma Aldrich). Using a magnetic stirrer, the dispersion was rapidly stirred for an hour before the addition of PGS and PCL (Mn 80 ​kDa; Aldrich, MO, USA). The PGS, PCL, and the modified BGs were completely dissolved after 4 ​h of stirring the solutions, and the weight ratio of PGS, PCL and the modified BGs was determined to be 1:1:0.1 (w/w). Following the addition of 0.25% glutaraldehyde as a cross-linking agent, the mixture was stirred for 24 ​h. The solvent was removed from the solutions by heating them to 50 ​°C and then granulating them for use as ink in an extrusion-based 3D bioprinter (3DPL BIOPRINTER N2, Iran). The small-diameter vascular grafts were designed in Rhinoceros® CAD software (version 5 SR8, Robert McNeel & Associates) in a porous tubular shape, with height, internal diameter, wall thickness, pore size, and infill percentage set to 10 ​mm, 5 ​mm, 0.3 ​mm, 300 ​μm, and 70%, respectively, and saved in. Stl file format. The G-code files were then sliced using CURA slicing software (Ultimaker BV, Geldermalsen, Netherlands). Next, the porous vascular grafts were prepared using G-code files imported into the 3D printer. At 80 ​°C, the granules were liquefied. The molten PCL, PGS, and modified BGs strands were then applied layer-by-layer to a 5 ​mm diameter rolling rod to form a patterned 45°/-45° porous structure. The five types of 3D printed vascular grafts prepared using the 3D printer were P: P: B0 (PCL-PGS-B0), P: P: B1 (PCL-PGS-B1), P: P: B2 (PCL-PGS-B2), P: P: B3 (PCL-PGS-B3), and P: P (PCL-PGS) as a control graft. The grafts were immersed in distilled water for three days after the inner layer was printed, and then washed three times with ethanol to remove any unreacted glutaraldehyde and PGS [28].

**Fig. 1:**
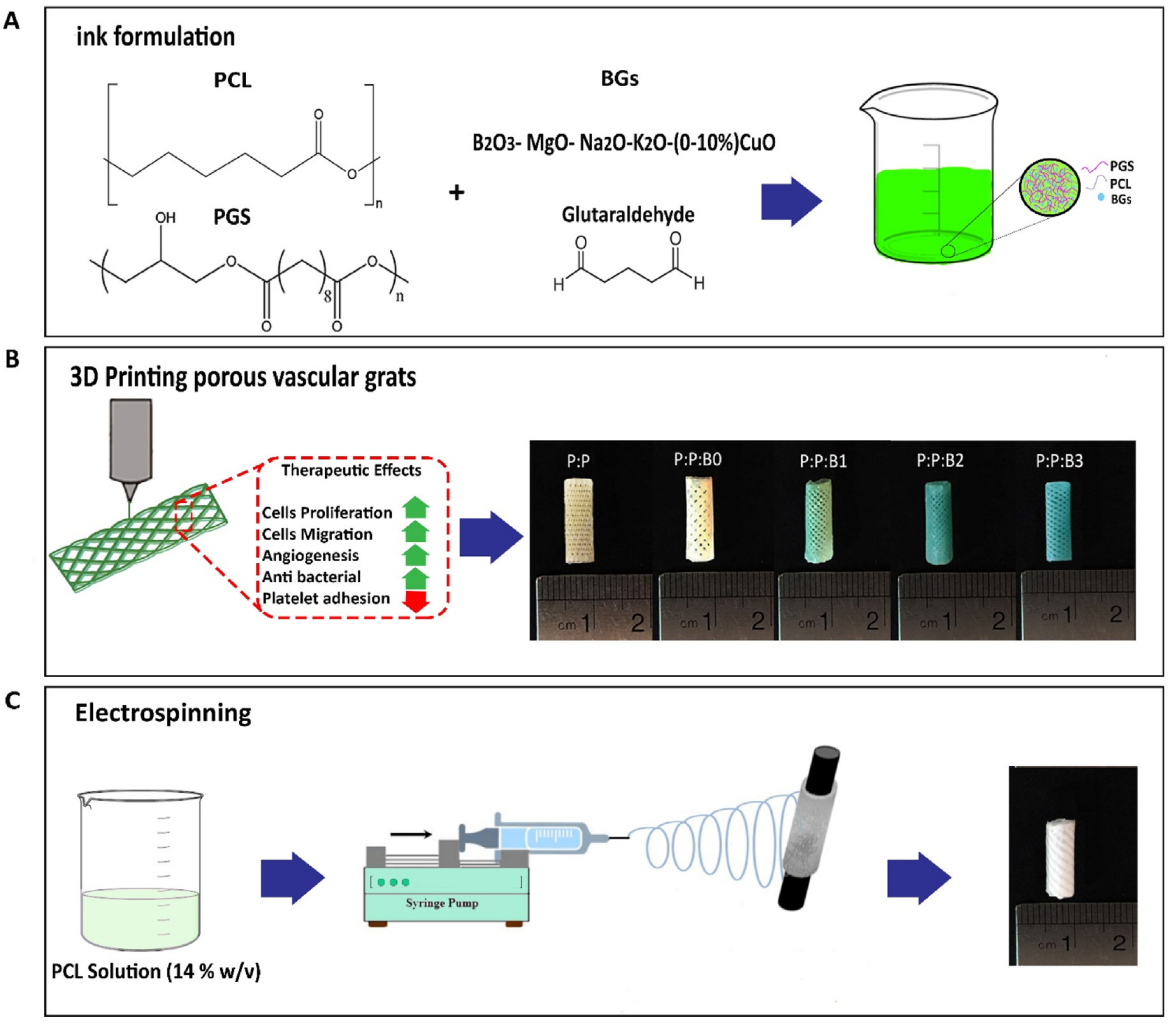
Schematic representation of sequential steps of manufacturing bilayer vascular grafts. (A) Preparation of ink containing PCL, PGS, and various compositions of the modified BGs. (B) Fabrication of the first layer of vascular grafts by 3D-printing technique, their therapeutic effects, and macroscopic side view images of 3D-printed P:P and P:P:Bs vascular grafts. (C) Fabrication of the second layer of vascular grafts by electrospinning technique and macroscopic side view image of the vascular graft.

The 3D-printed grafts were left to dry on aluminum foil for the bilayer graft to be fabricated, and it was then employed as a collector in the electrospinning process. In this study, a 7:3 (weight ratio) mixture of PCL in dichloromethane and methanol (Sigma Aldrich) was used to obtain a 14% w/v solution and electrospun onto a rotating P:P and the P:P:Bs template at 120 ​rpm. The procedure for fabricating bilayer vascular grafts are schematically shown in Fig. 1. The compositions of the modified BGs and the different groups of vascular grafts abbreviations are listed in Table 1.

### 2.3 Characterization of the modified BGs

To determine the crystal structure of the modified BGs, X-ray diffraction (XRD) (Philips PW1700 series Automated Powder Diffractometer) with a Cu−Kα radiation in 2θ range of step size 0.04° was employed. X-ray Fluorescence (XRF) (ThermoScientific, USA) was used to determine the chemical composition of the modified BGs. The critical temperatures of the modified BGs were determined using a technique known as simultaneously thermal analysis (STA) (BÄHR – STA 503, Germany). In order to obtain critical temperatures such as glass transition temperature ($T_{g}$) and peak crystallization temperature ($T_{c}$) during DTA analysis, the modified BGs powder was heated to 1200 ​°C from room temperature with the rate of 10° C/min.

### 2.4 Characterization of vascular grafts (FTIR and SEM)

In order to determine the chemical composition of vascular grafts before and after the cross-linking process, Fourier Transform Infrared spectroscopy (FTIR) with a range of 400–4000 ​cm^−1^ wave number was employed for the P:P and the P:P:Bs vascular grafts, using a VECTOR33 spectrometer to control PGS, PCL, and the modified BGs interactions. The surface morphology and compositional analysis of synthesized different groups of vascular grafts were characterized by scanning electron microscopy (SEM) (VEGA, TESCAN-XMU, Kohoutovice, Czech Republic) equipped with energy-dispersive X-ray spectroscopy (EDS) mapping, after coating with a thin layer of gold. Viscosity measurements were performed using an MCR-301 rheometer. At 25 ​°C, the P:P and P:P:Bs solutions were prepared at a 40% w/v concentration.

### 2.5 Degradation measurements of vascular grafts

Vascular grafts were shaped into small cylindrical pieces (5 ​× ​2 ​mm^2^), dried overnight in an oven at 50 ​°C, weighted, and then incubated at 37 ​°C soaked in phosphate-buffered saline (PBS) for a total of 60 days to evaluate weight loss as a function of time. Three sample pieces were taken out at precise time periods, rinsed with deionized water to eliminate any degradation remains three times, and dried at 50 ​°C for 24 ​h before being reweighted and immersed in degradation solutions again. Enzymatic degradation was also performed to evaluate vascular graft degradation *in vivo*. Each vascular graft was placed in 2 ​ml of PBS that contained 1 ​mg/ml of lipase (Lipase from porcine pancreas, Molekula, UK) and then incubated at 37 ​°C. Degradation media were replaced every 3 days. The percentage of weight loss was determined at predetermined intervals. Eq. (1) was used to determine the degradation percentage of each vascular graft [29]:

(1) $$Degradation\;\left(\%\right)=\frac{m_{0}-m_{t}}{m_{0}}\times 100$$

The initial mass and the mass of grafts at each time point, are m_0_ and m_t_, respectively.

### 2.6 Acidity measurement of vascular grafts immersed in culture medium

A pH measurement was taken to examine how the pH of the culture medium changed after adding synthesized vascular grafts. In summary, 0.4 ​g of each P:P graft and P:P:Bs graft were sterilized, and immersed in 4 ​ml of serum-free Dulbecco-modified eagle medium (DMEM, Gibco, USA). Afterward, test tubes were put into a conventional tissue culture incubator with 5% CO_2_ at 37 ​°C. The pH changes were recorded daily by inserting a pH meter (Hanna, Italy) into the solutions. The pH was measured on day 0 following a 4-h incubation of the P:P and the P:P:Bs grafts in the medium [30]. Every three days, the culture medium was replaced to mimic typical tissue culture protocols.

### 2.7 Elemental analysis of ions released from vascular grafts

To determine the concentration of ions released into culture media from vascular grafts containing the modified BGs, ICP-AES was employed. Each test tube received 100 ​mg/ml of the P:P:Bs grafts and the testing time was approximately 120 ​h in static mode. The length of testing periods was chosen when signals showed no additional significant increase or decrease. Sterilized P:P:Bs grafts were statically immersed in serum-free DMEM and incubated for up to 5 days at a constant 37 ​°C cell culturing environment, and the medium was collected at each time point. Then, using deionized water, it was diluted 10-to 20-fold. Finally, elemental analysis was conducted using inductively coupled plasma atomic emission spectrometry (Perkin-Elmer Optima 7000DV).

### 2.8 Mechanical characterization

Three vital characteristics of synthetic vascular grafts—modulus, Young's elongation, and stress at the break—were measured using a mechanical loading test in the axial direction on a uniaxial Z0.5 test machine (Zwick Roell, Ulm, Germany). Every tubular graft was cut into rectangular sheets with a 5 ​mm width and a 30 ​mm height. Using a digital caliper, the thickness of each vascular graft was measured (Series 209, Mitutoyo, USA). With the use of a 100 ​N load cell, the grafts were fixed in the tensile tester. At a rate of 10 ​mm/min, stress was used until rupture. The machine assessed the stress/strain on the grafts along their diameter and reported the results. Each examination was carried out on three grafts of separately made tubes to provide statistically significant outcomes. In addition to this, the potential of vascular grafts for suture retention was evaluated. Following the aforementioned procedures, a single loop of 3–0 polydioxanone suture was made about 2 ​mm from one free end of each vascular grafts. Each end of the grafts and the suture loop were fastened to the grips of the tensile machine. Here, a suture extension rate of 1 ​mm/s was employed to pull the suture, and the retention strength of the suture was determined as the highest force that was applied during the experiment. In this research, a demonstration of the mean ​± ​standard deviation values is used for all quantitative data reported.

### 2.9 Swelling measurement and cross-linking density determination

Crosslinking density of the polymeric matrix was determined by measuring swelling percentage in tetrahydrofuran (THF, Merck) at room temperature, according to previously published procedures [31]. First, dried vascular grafts are weighted and placed in THF. The vascular grafts were then taken out of the solvent for each day and weighed in a sealed vial to calculate how much swelling had occurred after being swabbed with filter paper to remove any remaining solvent on the surface. This process will continue until the grafts are considered to be fully swollen, which is typically three days. This mass is regarded as the mass of an equilibrium swollen network ($m_{eq}$). Finally, grafts were dried at room temperature using a vacuum oven for 5 days and weighted to calculate the dried network ($m_{d}$). Eq. (S1) shows the calculation of swelling percentage [30]. Eq. (S2) shows the volume fraction of polymer at equilibrium swelling ($\nu_{2}$). For calculating network strand density of swollen materials (ν), Flory-Rehner expression for the tri-functional affine network was employed [30]. The cross-linking density of an elastomeric material can also be calculated through E as follows [32]. Therefore, by comparing the extracted values from the mechanical tests, Eq. (S3), and Eq. (S4), the Flory-Huggins parameter is calculated [31].

### 2.10 In vitro experiments

#### 2.10.1 Leachate collection from vascular grafts

To produce the vascular graft leachates, based on ISO 10993–5:2009 test, 0.4 ​g of sterilized vascular graft were maintained in 4 ​ml of DMEM medium at 37 ​°C [6,30]. After 24 ​h, grafts were withdrawn from the solution and stored in a refrigerator. Fresh 10% fetal bovine serum (FBS) and 1% penicillin-streptomycin (P/S) solutions were added into the leachates before the assays.

#### 2.10.2 Cell culture and morphological study of HUVECs

In DMEM consisting of 10% FBS, 1% P/S antibiotic solution and 0.03 ​mg/ml endothelial cell growth supplement (ECGS, E2759- Sigma Aldrich), HUVECs were cultured at 37 ​°C and humidified air containing 5% CO_2_. Using trypsin, a confluent monolayer of HUVECs was removed from the flask, and trypan blue staining was used to determine the number of viable cells. HUVECs were seeded at 10^4^ ​cells/well in a 96-well plate and then leachates from vascular grafts were added to each well (n ​= ​3). After 24 ​h incubation at 37 ​°C and 5% CO_2_, a fluorescent microscope (Olympus, Japan) was used to examine the effects of leachates from vascular grafts on the proliferation and morphology of HUVECs using live-dead staining.

#### 2.10.3 Cell viability

MTT colorimetric assays were performed on days 1, 3, and 7 post-cell seeding to measure cell viability under the influence of various groups of vascular grafts. Following seeding the 96-well plate with 100 ​μl of HUVECs suspension (5 ​× ​10^3^ ​cells/well) on the sterilized vascular grafts that were supplemented with 10% FBS and 1% P/S, the plate was placed in an incubator at 37 ​°C and 5% CO_2_. Following the removal of the medium at each time point, 100 ​μl of MTT ((3,4-dimethylthiazol-2-yl)-2,5-diphenylte tetrazolium bromide) in PBS was added to each well (n ​= ​3). After 4 ​h of incubation at 37 ​°C and 5% CO_2_ in the dark, the supernatant was replaced with 100 ​μl of DMSO and shaken for 15 ​min to dissolve the formazan crystals that were generated by living cells during the incubation. A microplate reader (Bio-Tek, USA) was used to determine the absorbance at 570 ​nm after transferring 100 ​μl of the supernatant to a clean 96-well plate.

#### 2.10.4 Cell migration assay

Effective endothelialization of a vascular graft depends on the rate at which ECs migrate from adjacent native endothelium. The impact of vascular graft composites and control graft on the rate of ECs migration was examined using scratch assays. To obtain a confluent monolayer, HUVECs were seeded, at the density of 10^4^ ​cells per well, in 48-well plates (n ​= ​3). In this study, mitomycin-C was used to ensure that the cells migrated and did not proliferate. Before to performing the scratch test, 250 ​μl of 5 ​μg/ml mitomycin-C in culture medium was added to each well and incubated at 37 ​°C and 5% CO_2_ for 2 ​h. After removing the culture medium containing mitomycin-C, a 100 ​μl pipette tip was used to generate a vertical scratch wound, and then cells were washed with PBS to remove unattached cells [33]. Since the migration of cells from adjacent vessels is essential for endothelialization, the scratch line is a cell-free area that might simulate the borders of the graft after implantation. The wounded monolayers were incubated with fresh culture medium (positive control), control graft leachate, or the P:P:Bs vascular graft leachates for 24 ​h at 37 ​°C. Cell migration was observed at 0 and 24 ​h following scarification using an inverted microscope (Olympus, Japan).

#### 2.10.5 ELISA assay for VEGF secretion

The secretion of the VEGF protein is one of the defining characteristics of angiogenesis. For the VEGF assay, HUVECs were seeded at a density of 2 ​× ​10^4^ ​cells/well in 24-well plates on the different groups of vascular grafts. The culture medium was replaced on the third day of the culture. On the fifth day, the VEGF ELISA kit (Cusabio Systems) was used to carry out the VEGF ELISA in accordance with the kit's instructions. The amounts of VEGF were determined using a standard curve, and the results were expressed as the amount of VEGF (pg/ml) of supernatant.

#### 2.10.6 Graft-bacterial interaction experiment

The gram-negative and gram-positive strains of bacteria were selected to study the impact of modified BGs incorporated in vascular grafts on bacteria. Single bacterial colonies of *Staphylococcus aureus* (*S. aureus,* ATCC 25923) and *Pseudomonas aeruginosa* (*P. aeruginosa,* ATCC 27853) were cultured in 10 ​ml of tryptic soy broth (TSB) and lysogeny broth (LB), respectively, at 37 ​°C overnight. After washing with sterile PBS and vortexing, the optical densities (OD) of overnight cultures were measured with a spectrophotometer (Lambda 25, PerkinElmer, Milan, Italy) at 600 ​nm and fixed to 10^7^ ​CFU ​ml^−1^. The P:P and P:P:Bs grafts were cultured in 2 ​ml of the bacterial solutions at 37 ​°C under static conditions and in the dark.

At the specified time intervals of 3, 6, and 24 ​h, aliquots from each tube were taken at the specified time intervals, and to count colonies, they were consecutively diluted in sterile PBS on nutrient agar plates. Six different experiments were conducted. Additionally, a disc diffusion test was used to examine the antibacterial activity of P:P and P:P:Bs vascular grafts. *P. aeruginosa* and *S. aureus* solutions were prepared and homogeneously swiped on nutrient agar plates (n ​= ​3), at 10^5^ ​CFU ​ml^−1^ concentration. UV-sterilized grafts were placed on the plates and incubated at 37 ​°C, after inoculation. Using a camera, the inhibition zones were examined and photographed after 24 ​h of incubation.

#### 2.10.7 Hemocompatibility

##### 2.10.7.1 Quantification of whole blood clotting time

In order to evaluate the anticoagulant property of vascular grafts, a whole blood kinetic clotting time technique was employed. Ethylenediaminetetraacetic acid (EDTA) anticoagulant vacutainer tubes were used to collect blood from healthy adult participants after venipuncture. To avoid tissue thromboplastin contamination, which is a result of needle penetration, the first 3 ​ml of blood were eliminated. Square grafts (64 ​mm^2^) were used for each time point. In summary, 850 ​μl of CaCl_2_ (0.1 ​M) was added to the 8.5 ​ml of EDTA blood to initiate the clotting process. A 25 ​μl amount of activated blood was carefully applied to the wells of a 24-well plate for the P:P:Bs grafts, control graft, and blank well. All grafts were incubated for 5, 15, 25, 35, 45, and 60 ​min at room temperature. After each timepoint, the grafts were incubated in 750 ​μl of distilled water for 5 ​min. After adding distilled water, red blood cells (RBC) that were not entrapped in the thrombus were lysed, releasing hemoglobin into the water for subsequent measurement. Absorbance at 540 ​nm was measured, using a 96-well plate reader, to determine the amount of hemoglobin present in the solution. The absorbance value is negatively associated with the size of the clot.

##### 2.10.7.2 Erythrocyte hemolysis ratio

The use of implantable biomaterials, particularly vascular prostheses, is significantly influenced by hemocompatibility [34]. Each graft was divided into 25 ​mm^2^ squares for the hemolysis test and washed with distilled water 3 times. The vascular grafts were then placed in deionized water or a solution of 0.9% NaCl to perform a hemolysis test. Following this, 0.1 ​ml of fresh human blood was added to the mixture. After 30 ​min, erythrocyte membranes ruptured and hemoglobin was discharged into the solution. After 30 ​min of incubation, the supernatants from each vascular graft were collected and centrifuged at 3000 ​rpm for 5 ​min. The absorbance at 540 ​nm was determined by spectrophotometer after the supernatants had been collected and put in cuvettes. Positive and negative controls were prepared by mixing 100 ​μl of fresh blood with 5 ​ml of distilled water and 0.9% NaCl. The following equation was used to calculate the hemolysis percentage (HR):

(2) $$HR=\frac{As-An}{Ap-An}\times 100$$

where *A*s is the absorbance value of hemoglobin in the grafts and *A* and *A*p are absorbance values of hemoglobin in negative and positive controls, respectively.

##### 2.10.7.3 Platelet adhesion test

In order to morphologically evaluate platelet activity, the vascular grafts were allowed a 60-min immersion in 200 ​μl of fresh platelet-rich plasma (PRP). Following three washes in PBS, the grafts were fixed in a 2.5% glutaraldehyde solution at 37 ​°C overnight. The grafts were then dehydrated using an ethanol gradient and air-dried overnight at room temperature. Following the gold sputter coating, the morphology of the platelets was examined by SEM.

### 2.11 In vivo experiments

The Animal Ethics Committee of Iran Medical University gave its approval to all animal surgical procedures. Ten male mice (8 weeks old, weighing 25–30 ​g) were employed for the experiment. All surgical techniques on the animals were performed in conformity with the IR-approved animal research protocol (IR.IUMS.REC.1401.806) and the internationally recognized standards for the use of laboratory animals [35]. Ketamine/Xylazine was used to produce and maintain general anesthesia at a dose of 50 ​mg ​kg^−1^/10 ​mg ​kg^−1^. Prior to being draped for surgery, the mice were shaved and cleaned with iodine. A scalpel was used to make two tiny incisions on the middle dorsal surface of each mouse. A pocket was made in the subcutaneous area using blunt forceps. The P:P or P:P:Bs grafts were implanted into each subcutaneous pocket, and the sutures were used to close the incisions. The animals were given unrestricted access to food and water, and daily checks were performed for any possible problems. All animals were sacrificed 2 weeks following surgery, and the subcutaneous implants were taken out for histological evaluation. The subcutaneous implants were embedded in paraffin after being post-fixed in 4% paraformaldehyde for 24 ​h. A microtome was used to cut the blocks into 5 ​μm thick sections, which were then deparaffinized and rehydrated. The slides were then stained with hematoxylin and eosin (H&E), and the angiogenesis activity was assessed by viewing them through a digital pathology slide scanner microscope.

### 2.12 Tissue preparation

The vascular grafts and an adjacent 1 ​cm of normal skin were removed 4 weeks after subcutaneous implantation and then prepared for reverse transcriptase polymerase chain reaction (RT-PCR) analysis as described below.

#### 2.12.1 Real-time RT-PCR

cDNA was made from 1 ​μg of extracted total RNA from collected tissue samples using the Superscript first-strand synthesis system (Invitrogen). Real-time PCR was performed with 2X Q-PCR Master Mix (SYBR, Rox) (SMOBIO Technology, Hsinchu, Taiwan) and analyzed with ΔΔCT method and REST® 2009. RNAs from all tissue samples were extracted, and cDNAs were reverse transcribed from the total RNA. The relative expression of specific genes in angiogenesis (VEGF) and inflammation (tumor necrosis factor-alpha (TNF-α), and interleukin 1-beta (IL-1β)), was evaluated using 2X Q-PCR Master Mix (SYBR, Rox) (SMOBIO Technology, Hsinchu, Taiwan) and analyzed with ΔΔCT method and REST® 2009. Gene expression was normalized to the level of glyceraldehyde 3-phosphate dehydrogenase (GAPDH). The primers used in real-time PCR assays are described in Table 2.

**Table 2:** Sequences of primers used for the amplification of the genes through real-time RT-PCR technique.

Gene	Forward Sequence	Reverse Sequence
GAPDH	AAGGTGAAGGTCGGAGTCAAC	GGGGTCATTGATGGCAACAATA
IL-1β	***ATGATGGCTTATTACAGTGGCAA***	GTCGGAGATTCGTAGCTGGA
TNF-α	CCTCTCTCTAATCAGCCCTCTG	GAGGACCTGGGAGTAGATGAG
HBEGF	ATCGTGGGGCTTCTCATGTTT	TTAGTCATGCCCAACTTCACTTT
PDGFB	CTCGATCCGCTCCTTTGATGA	CGTTGGTGCGGTCTATGAG
VEGF	AGGGCAGAATCATCACGAAGT	AGGGTCTCGATTGGATGGCA

#### 2.12.2 Vascular formation assay

To determine if angiogenesis had developed around the scaffolds, immunofluorescent staining for endothelial cell markers (CD31) was performed for each group. Following overnight incubation at 4 ​°C with a rabbit polyclonal primary antibody to CD31 (Ab28364, 1:100, Abcam, USA), the samples were incubated with a goat anti-rabbit IgG H&L secondary antibody (1:50, Abcam, USA) at room temperature for 90 ​min. The nuclei were stained with DAPI dye (Sigma Aldrich, no. D9542, US). The density of capillaries was calculated by counting the number of VEGFR2-positive vascular structures in three randomly chosen fields/sections.

### 2.13 Statistical analysis

For statistical analysis, a one-way or two-way analysis of variance (ANOVA) was conducted, followed by Tukey or Bonferroni posttests, respectively, with P ​< ​0.05 indicating significance. All information was expressed as mean ​± ​standard deviation (SD).

## 3 Results and discussion

### 3.1 Characterization of the modified BGs

The characterization of modified BGs was discussed in our previous study in detail [27]. XRD patterns of as-prepared modified BGs showed large halos, indicating their amorphous structure. The modified BGs contained Cu in line with the nominal weight ratios and had no alumina or silicon contaminants, according to XRF analysis. In addition, the removal of calcium and phosphate components caused that none of the groups ruptured RBCs or activated the coagulation cascade, and the introduction of Cu ions to modified BGs significantly improved angiogenesis properties [27]. Accordingly, the synthesized modified BGs may be applied to vascular grafts without concern for coagulation.

### 3.2 Chemical characterization of vascular graft inks

**Fig. 2:**
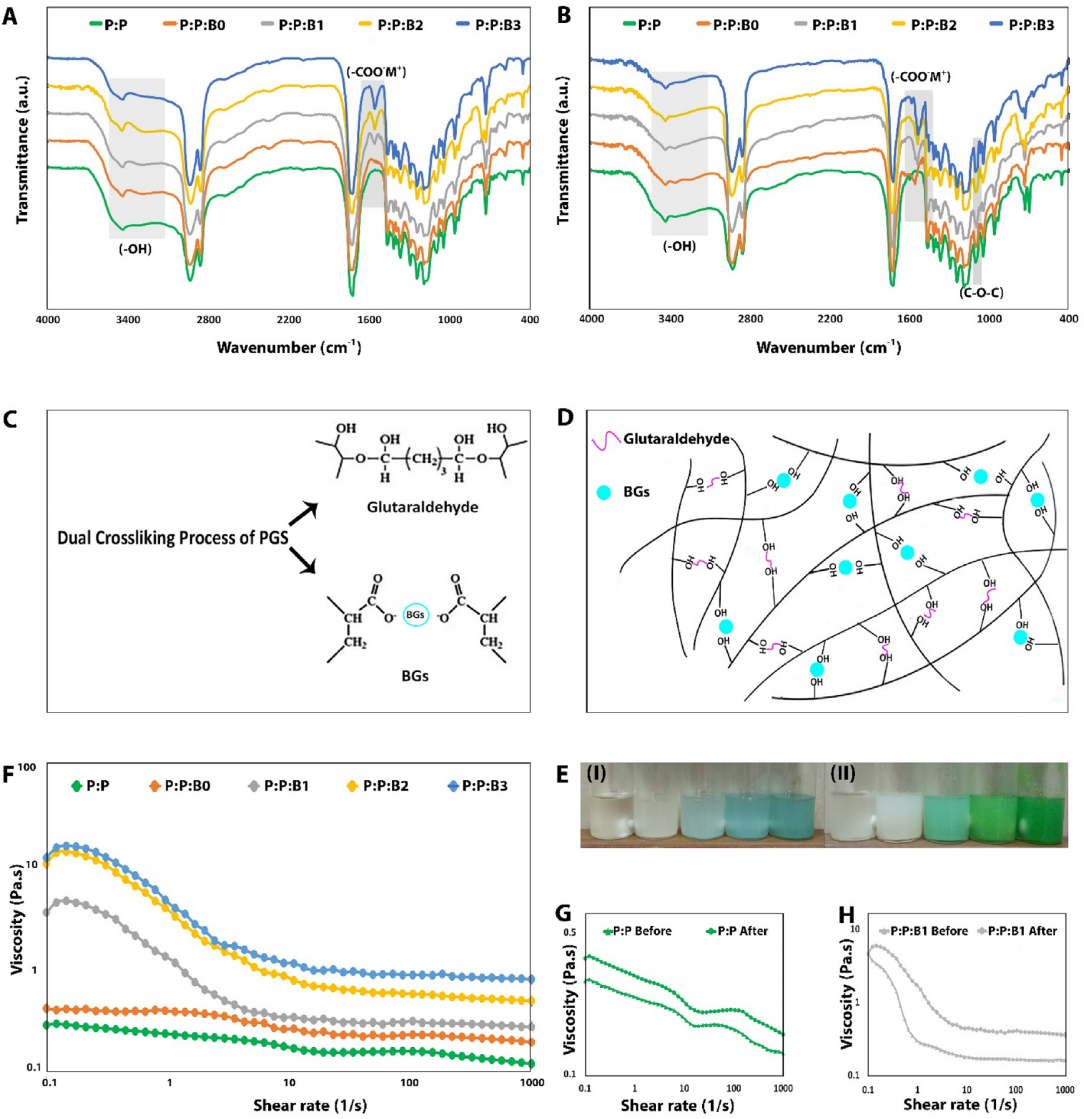
FTIR spectra of (A) before and (B) after the addition of glutaraldehyde into P:P and P:P:Bs solutions. Reduction of peak intensity observed related to –OH groups. New peaks were observed related to (-COO^-^M^+^) groups. An increase in peak intensity was observed related to *C*–*O*–C groups. (C, D) Schematic representation of the dual cross-linking process of PGS by the modified BGs and glutaraldehyde. (E) Variation of viscosity of P:P and P:P:Bs solutions before (I) and after (II) addition of glutaraldehyde. (F) Comparison of viscosity of P:P and P:P:Bs solutions after addition of glutaraldehyde. (G, H) Comparison of viscosity of P:P and P:P:B1 solutions, respectively, before and after the addition of glutaraldehyde.

Fig. 2 shows P:P and P:P:Bs composites FTIR spectra before and after the addition of glutaraldehyde. The PGS absorption bands overlap with PCL-related vibration bands, namely the alkyl group-related ones at 2938 ​cm^−1^, 2863 ​cm^−1^, the *C*–O band stretching vibration at 1165 ​cm^−1^, and the carbonyl stretching (C=O) peak at 1734 ​cm^−1^. In addition, the stretch vibration of the hydroxyl bond is responsible for the wide band in the range of 3300 and 2700 ​cm^−1^ [36]. All of the spectra include typical PCL bands that have been previously described in the literature [36], including asymmetric and symmetric *C*–*O*–C stretching peaks at 1165 cm-1 and 1240 cm-1, respectively, and backbone *C*–O and *C*–C stretching peaks at 1294 cm-1, as well as carbonyl stretching (C=O) peak at 1722 cm-1. The primary PCL bands overlap with several distinct bands associated with PGS and BG particles, making it difficult to see these bands in Fig. 2A. Additionally, BGs show peaks associated with the tetrahedral BO_4_ groups' B–O stretching mode that is located at 700 ​cm^−1^ and between 900 and 1100 ​cm^−1^, as well as 1150-1300 ​cm^−1^ and 1200-1500 ​cm^−1^ range bands for B–O stretching of BO_3_ group [37].

The FTIR spectra of the just mixed the P:P and P:P:Bs composites are shown in Fig. 2A. In the P:P:Bs composites' FTIR spectra, in the range of 1510 and 1650 ​cm^−1^, new peaks have been developed. These new peaks are the result of metallic carboxylate stretches of potassium, sodium, or magnesium carboxylates in B0 as well as the Cu carboxylates in B1, B2, and B3 [30]. This confirms that the unreacted carboxylic groups in the PGS pre-polymer, which were used to synthesize the P:P:Bs composites, hydrolyzed the ceramic during the synthesis of the grafts. It is conceivable that, in comparison to P:P counterpart, producing metallic carboxylation uses carboxylic groups and subsequently decreases OH bond (3300-2700 ​cm^−1^) in FTIR spectra.

Fig. 2B exhibits the FTIR spectra of the P:P and the P:P:Bs composites after 24 ​h of adding glutaraldehyde. It was confirmed that the presence of glutaraldehyde caused a decrease in the relative intensity of the OH bands and an increase in the peak at 1105 ​cm^−1^ related to C–O–C groups [38]. Also, the formation of metal carboxylate groups increased after 24 ​h. The FTIR spectrum of PCL and PGS before and after adding glutaraldehyde and the modified BGs revealed that PCL did not participate in the crosslinking reaction and that the modified BGs and glutaraldehyde exclusively interact with PGS. Therefore, there is conclusive proof that metal oxides in the modified BGs structure and glutaraldehyde interact with the carboxylic acid and hydroxyl groups in the PGS matrix. Firstly, FTIR spectrum between 1500 and 1600 ​cm^−1^ which is connected to metal carboxylate stretching absorptions showed that the modified BGs interacted with carboxyl groups before adding glutaraldehyde and produced metal carboxylates (-COO^-^M^+^) groups. The process of the PGS cross-linking related to the addition of glutaraldehyde and the modified BGs is clarified in Fig. 2C and D. Secondly, adding glutaraldehyde to P:P and P:P:Bs solutions notably raised the viscosity and changed the color of the solution (Fig. 2E), demonstrating a coupling of the branching pre-polymer PGS molecules by consumption of hydroxyls (the OH stretch at around 3300 ​cm^−1^ in reaction with glutaraldehyde and generating a cross-linked network at room temperature).

As suggested previously, glutaraldehyde and modified BGs are active in the PGS cross-linking process. During the preparation of ink, the viscosity of the P:P and P:P:Bs solutions following the addition of glutaraldehyde was measured quantitatively at 25 ​°C. After 24 ​h of cross-linking, the solutions' viscosity curves are displayed in Fig. 2F. The viscosity values of all cross-linked solutions (P:P and P:P:Bs solutions) were found to decrease gradually with increasing shear rates, as is typical of pseudoplastic fluids [39]. The addition of modified BGs led to an increase in the apparent viscosity of the solutions, which was related to the amount of Cu in the BGs' structure; therefore, the P:P:B3 solution demonstrated the greatest value. Fig. 2G and H illustrates the difference in viscosity between the P:P and P:P:B1 solutions before and after the addition of glutaraldehyde. Observations indicated that the addition of glutaraldehyde increased the viscosity of the solutions. Consequently, after the addition of the modified BGs and glutaraldehyde, the bonding between ions produced by the modified BGs, specifically Cu, and glutaraldehyde with PGS molecular chains increased. This resulted in stronger entanglement and resistance to the movement of the molecular chain. Since glutaraldehyde and Cu have a synergistic effect on the cross-linking of PGS, reducing the percentage of glutaraldehyde is necessary to maintain the P:P:B3 solution's viscosity close to that of the P:P:B2 solution; this is essential for the 3D-printing of P:P:B3 vascular grafts with dimensions and porosity comparable to other grafts.

### 3.3 Macro and microstructure of bilayer vascular grafts

In this research, 3D printing and electrospinning techniques were effectively used to fabricate bilayer vascular grafts. According to CAD data, an extrusion-based 3D printer produced the initial layer of vascular grafts utilizing PGS, PCL, and the modified BGs. The vascular grafts are made of a porous tube wrapped in a thin electrospun PCL sheath to stop bleeding after implantation in accordance with the design parameters. The theoretical internal diameter (i.d.) for all grafts, was 5 ​mm, where actual i.d. Was 5.27 ​± ​0.063 ​mm, 5.32 ​± ​0.024 ​mm 5.29 ​± ​0.042 ​mm, 5.37 ​± ​0.021 ​mm and 5.36 ​± ​0.044 ​mm for P:P, P:P:B0, P:P:B1, P:P:B2 and P:P:B3 graft, respectively. All vascular grafts were printed by a nozzle 0.3 ​mm in size and the average wall thickness was 0.567 ​± ​0.021 ​mm, 0.472 ​± ​0.034 ​mm, 0.447 ​± ​0.022 ​mm, 0.411 ​± ​0.014 ​mm, 0.409 ​± ​0.01 ​mm for P:P, P:P:B0, P:P:B1, P:P:B2 and P:P:B3 graft, respectively.

**Fig. 3:**
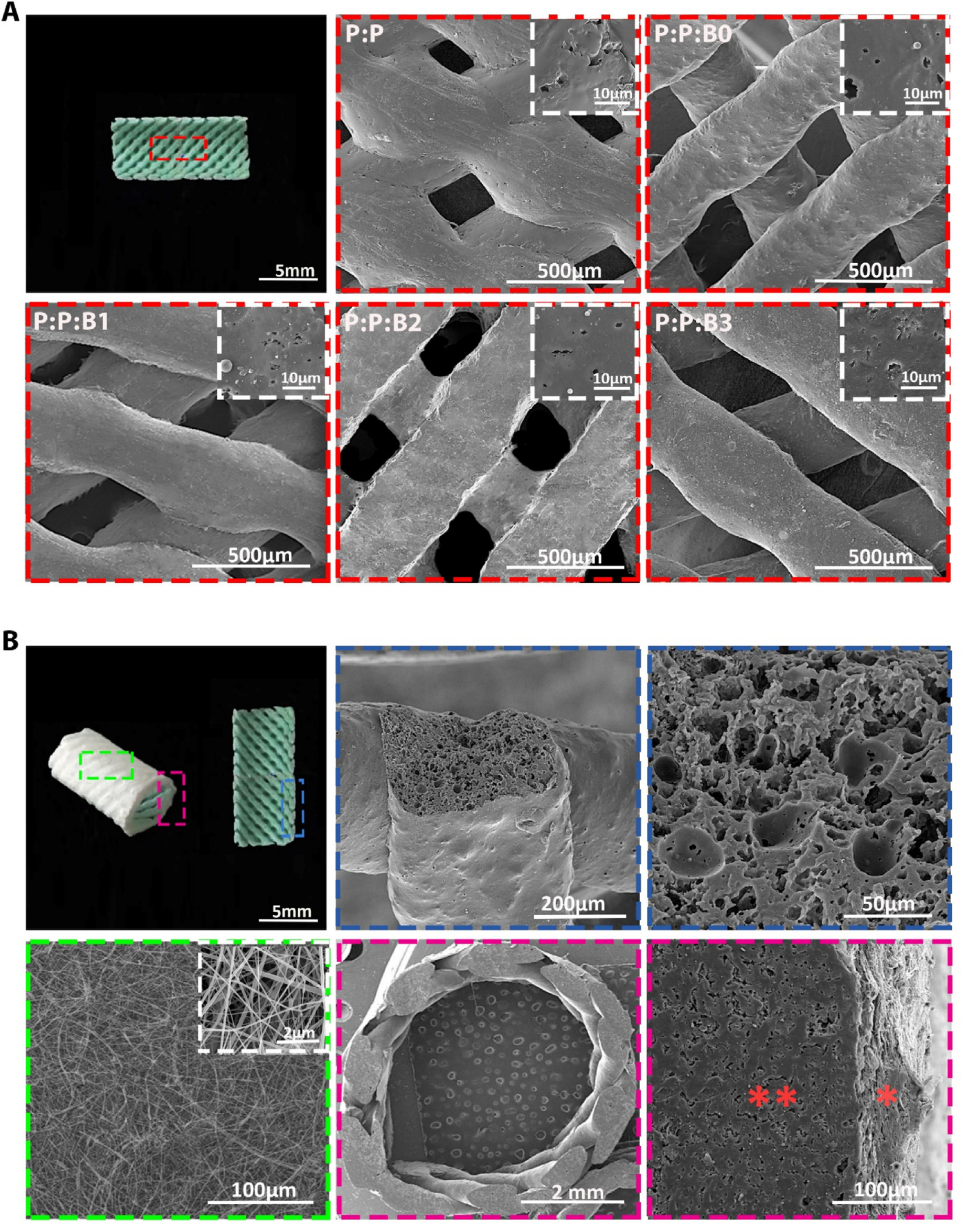
Fabricated bilayer vascular grafts' SEM image. (A) Macroscopic image from the luminal surfaces of vascular grafts, with a designated area matching the region of SEM images. SEM image of the luminal surface of vascular grafts (Red dotted boxes). (B) Macroscopic image from the border region of longitudinal sections and from adventitial surfaces of vascular grafts, with a designated area matching the region of SEM images. SEM image of the electrospun PCL sheath (Green dotted box). Cross section of the 3D-printed vascular graft strut (Blue dotted boxes). SEM image of a bilayer vascular graft top view and interface of 3D-printed and electrospun layers (Pink dotted boxes), ∗∗ indicates 3D-printed layer and ​× ​indicates electrospun layer. (For interpretation of the references to colour in this figure legend, the reader is referred to the Web version of this article.)

SEM was employed to evaluate the morphology of luminal and adventitia surfaces of the bilayer vascular grafts (Fig. 3). SEM analysis of the P:P:Bs grafts showed that the modified BG microparticles were evenly dispersed throughout the polymeric matrix with no discernible agglomeration. This demonstrates the reliability of the method used to fabricate vascular grafts containing 10 ​wt% modified BGs and verifies that measurements of the materials' properties were repeatable (Fig. 3A). The first layer of the vascular grafts' cross-section revealed that the 3D-printed struts had a porous structure that could facilitate cell infiltration from the nearby endothelium (Fig. 3B). The first layer of all vascular grafts was printed by a 0.3 ​mm nozzle, but the strut dimension after print was 0.61 ​± ​0.027 ​mm, 0.47 ​± ​0.044 ​mm, 0.45 ​± ​0.036 ​mm, 0.43 ​± ​0.027 ​mm, and 0.44 ​± ​0.012 ​mm for P:P, P:P:B0, P:P:B1, P:P:B2, and P:P:B3 grafts, respectively.

A sheath of PCL electrospun fibers was applied to the 3D-printed vascular grafts. The electrospinning procedure was carried out directly on the 3D-printed vascular grafts to generate strong adhesion between layers. Fig. 3B illustrates the morphology of the electrospun sheath; the developed network was free of flaws or beads. As illustrated in Fig. 3B, the adhesion between the 3D-printed layer and the electrotyping layer has occurred successfully, and there is no separation between the first and second layers. To promote adhesion between the inner and outer layers of vascular grafts, two approaches were used. There is evidence in the literature that slow solvent evaporation is responsible for the fusion of adjacent PCL fibers [40]. Solvent traces in fibers were essential for uniformly covering pores in the 3D grid and improving adhesion between the 3D-printed layer and the electrospun sheath. The low fiber stretching and jet elongation caused by the low voltage (10 ​kV) used to extrude the fibers may have slowed solvent evaporation until fiber collection. As fibers reached the 3D-printed vascular grafts, the trapped solvent continued to diffuse out before completely solidifying, which improved adhesion between the two layers of the synthetic vascular grafts [40]. Moreover, surface porosity is generated in the 3D-printed vascular grafts after immersion in ethanol and water (Fig. 3A), which might enhance the physical adhesion of the two layers. The distribution of B, Mg, Na, K, and Cu ions in the P:P:Bs vascular graft was examined quantitatively using 10.13039/100004679EDS mapping, and it was discovered that these ions were evenly distributed throughout the polymer matrix (the 10.13039/100004679EDS mapping shown in Fig. S1, in the Supporting Information, was only available for the P:P:B2 graft strut cross-section because the images of the 10.13039/100004679EDS mapping of the P:P: Bs graft strut cross-section were similar).

### 3.4 Analysis of the degradation behavior of vascular grafts

It is important to note that while both PGS and PCL are biodegradable polyesters, their degradation rates are rather different. PCL degrades more slowly; it has been shown *in vivo* that PCL-based implants can last up to two years, whereas PGS is resorbed in the body within 60 days [36]. As a result, the degradation behavior of P:P and P:P:Bs grafts was assessed *in vitro*, as mixing PCL and PGS with the different modified BGs compositions would alter the degradation rate of the mixture. With the P:P graft employed as control, the investigations were carried out for up to 60 days in PBS under static circumstances at 37 ​°C.

**Fig. 4:**
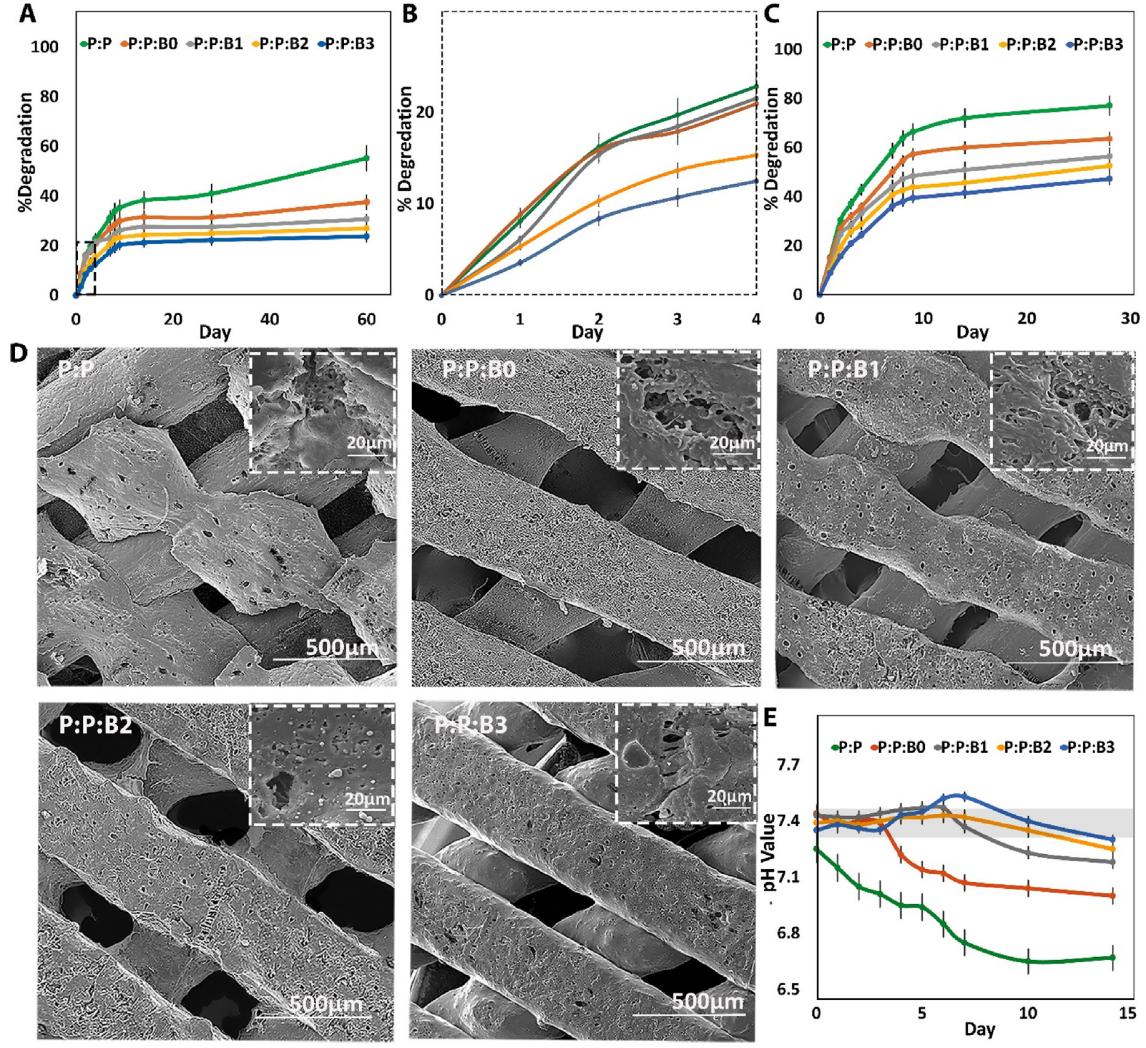
The degradation rate of synthesized vascular grafts over 60 days. (A) Weight loss percentage in PBS at 37 ​°C on a logarithmic scale, which was revealed to be linear degradation. (B) Weight loss percentage of grafts in the first 4 days of degradation test in PBS at 37 ​°C. (C) Weight loss rates of grafts during enzymatic degradation *in vitro*. (D) Representative SEM images of the vascular grafts degradation after 60 days incubation in PBS at 37 ​°C ​(E) pH value versus incubation time. Gray box indication of the normal physiological level (pH between 7.35 and 7.45).

Weight loss of the vascular grafts was recorded over a period of 3 months incubation in PBS (Fig. 4A and B). P:P:B0 and P:P:B1, P:P:B2, and P:P:B3 grafts had an approximately similar degradation profile after 60 days, with 37.6%, 30.7%, 27%, and 23.07% weight loss, respectively, whereas the P:P graft had a weight loss of 55.28%. After incubation in PBS, the morphologies of P:P and P:P:Bs grafts were evaluated by SEM and shown in Fig. 4B. Fractures and cracks were seen on the surfaces of P:P grafts after 60 days of incubation, while dotted morphology was visible on the surfaces of P:P:B0, P:P:B1, and P:P:B2 grafts (Fig. 4D). Also, similar morphologies were found on the surfaces of P:P:B3 graft. Release of unreacted carboxylic groups, hydrolysis of PGS, and dissolution of modified BGs microparticles are believed to be responsible for the degradation profiles of P:P and P:P:Bs grafts [40]. Consequently, the rate of degradation of the grafts is determined by the breakdown of PGS ester bonds in sebacic acid and glycerol monomers inside the composites. Therefore, the higher cross-link density led to longer degradation periods. According to FTIR results, the introduction of glutaraldehyde and modified BGs (especially those containing Cu) resulted in the cross-linking of the PGS matrix and prolonged degradation time. On the other hand, PGS degradation induces acidity in the surrounding environment, which accelerates the rate of hydrolysis [36]. However, the P:P:Bs grafts significantly influenced the pH values of the medium, resulting in a slower rate of degradation of the P:P:Bs grafts [41]. This was caused by the ability of the modified BGs to neutralize acidity, which explained why P:P:Bs grafts degrade at a slower rate than P:P grafts. Nevertheless, the degradation rate increased for the first three days in P:P:B0 and P:P:B1 grafts compared to P:P grafts due to the dissolution of the modified BGs; even so, this rate was decreased by increasing the fraction of Cu in vascular grafts.

Enzymatic degradation experiments mimicked a natural degradation condition by using lipase isolated from porcine pancreatic tissue. After 28 days of enzymatic degradation, there were significant reductions in mass for all the grafts (Fig. 4C). The rates of enzyme degradation were as follows: 77.1% for P:P, 63.6% for P:P:B0, 56.4% for P:P:B1, 52.6% for P:P:B2, and 47.3% for P:P:B3. In comparison to hydrolytic degradation, an enzymatic degradation test conducted *in vitro* was much more representative of the objective fact that occurs *in vivo*. It could provide more noticeable results in less time and offer a more effective comparison. Moreover, the *in vivo* environment is complex. Because there are many different types of enzymes, like esterase and lipases, that are able to degrade the substance, the enzymatic degradation experiment could more closely approximate the process of biodegradation that occurs *in vivo*. The biodegradation rate *in vivo* was likely higher than that obtained *in vitro*.

### 3.5 pH and ion concentrations in culture media during vascular grafts incubation

To identify the chemical composition of the P:P and P:P:Bs degradation byproducts, the pH of the incubation medium was utilized. Fig. 4E depicts a typical pH profile of 14 days of incubation of vascular grafts in the medium. The results demonstrate that the P:P graft had the highest pH reduction compared to the P:P:Bs grafts. The hydrolysis of PCL and PGS ester bonds leads to the release of carboxylic groups during ester cleavage and the presence of unreacted carboxylic acid groups on the PGS backbone. Both of these things contribute to the high acidity of the degradation medium [42]. Because of the acidic nature of the degradation, PGS can be cytotoxic, limiting its use in the human body [43]. The media in contact with the P:P graft, has a considerably lower pH value than the control media, the media without any vascular grafts, indicating that acidification had happened (Fig. 4E). The acidification was neutralized when the PGS and PCL contained alkaline the modified BGs, especially those containing Cu, with pH values maintained at around physiological levels during the 7-day incubation period. The P:P:B0 vascular graft was less successful in neutralizing the acidity of the medium after 4 days of incubation than the other P:P:Bs grafts. It is hypothesized that the P:P graft acidifies the culture medium by the ionization of carboxylic acid groups formed by the hydrolysis of the PGS ester (-COOR) groups and unreacted carboxylic acid (-COOH) groups in the PGS, as illustrated in the following equation [30]:

$$-COOH+H_{2}O\to -COO^{-}+H_{2}O^{+}$$

$$-COOR+2H_{2}O\to -COO^{-}+ROH+H_{3}O^{+}$$

In the case of the P:P:Bs grafts in culture media, the Na_2_O, K_2_O, MgO, and CuO react with water, generating sodium, potassium, Mg, and Cu hydroxide, which subsequently penetrate the polymeric matrix and in order to form metallic carboxylate, will react with carboxylic acid groups. The following reactions result in an increase in medium pH (i.e., alkalization) [30]:

$$-COOH+NaOH\to -COO^{-}Na^{+}+H_{2}O$$

$$-COOH+KOH\to -COO^{-}K^{+}+H_{2}O$$

$$2-COOH+Cu{\left(OH\right)}_{2}\to {\left(-COO^{-}\right)}_{2}Cu^{++}+2H_{2}O$$

Previous research indicated that when ECs were exposed to an acidic pH, cell proliferation and migration were slowed. Furthermore, in acidic conditions, VEGF was ineffective on ECs and did not activate downstream signaling pathways like AKT. The expression of VEGFR2 by endothelial cells was also dramatically suppressed at the molecular level by acidity [44]. Consequently, the production of an acidic environment by vascular grafts during the reendothelialization process can hinder the healing process; thus, in this research, the addition of modified BGs to vascular grafts could be effective in neutralizing acidity and the regeneration process.

The percentage of B and Cu released from P:P:Bs grafts into the medium was evaluated. Results of B release from P:P:Bs grafts into the solution were gradual, as shown in Fig. S2 in supporting information. Without a burst release, releasing period of the B from all vascular grafts was longer than 5 days, which leads to slower ion diffusion into the culture medium. The greater release of B in the P:P:B3 graft in comparison to the other grafts is related to the rapid degradation of B3, which is consistent with the DTA findings that were analyzed in our previous study [45]. The cumulative percentage of Cu released into the culture medium was also identical to B, where the release was increased slowly with no release burst. For the Cu-releasing vascular grafts, the percentage of Cu released from the grafts increased with time over 5 days. With the exception of the first half hour of immersion, when the P:P:B1 graft showed slightly more Cu-released relative to the P:P:B2 and P:P:B3 grafts, the percentage of Cu released from the grafts increased with increasing Cu in the modified BGs at any immersion period. The cumulative percentage of Cu that was released from the grafts over the period of five days was 53.7% for the P:P:B1 graft, 66.1% for the P:P:B2 graft, and 82.5% for the P:P:B3 graft.

### 3.6 Mechanical properties

**Fig. 5:**
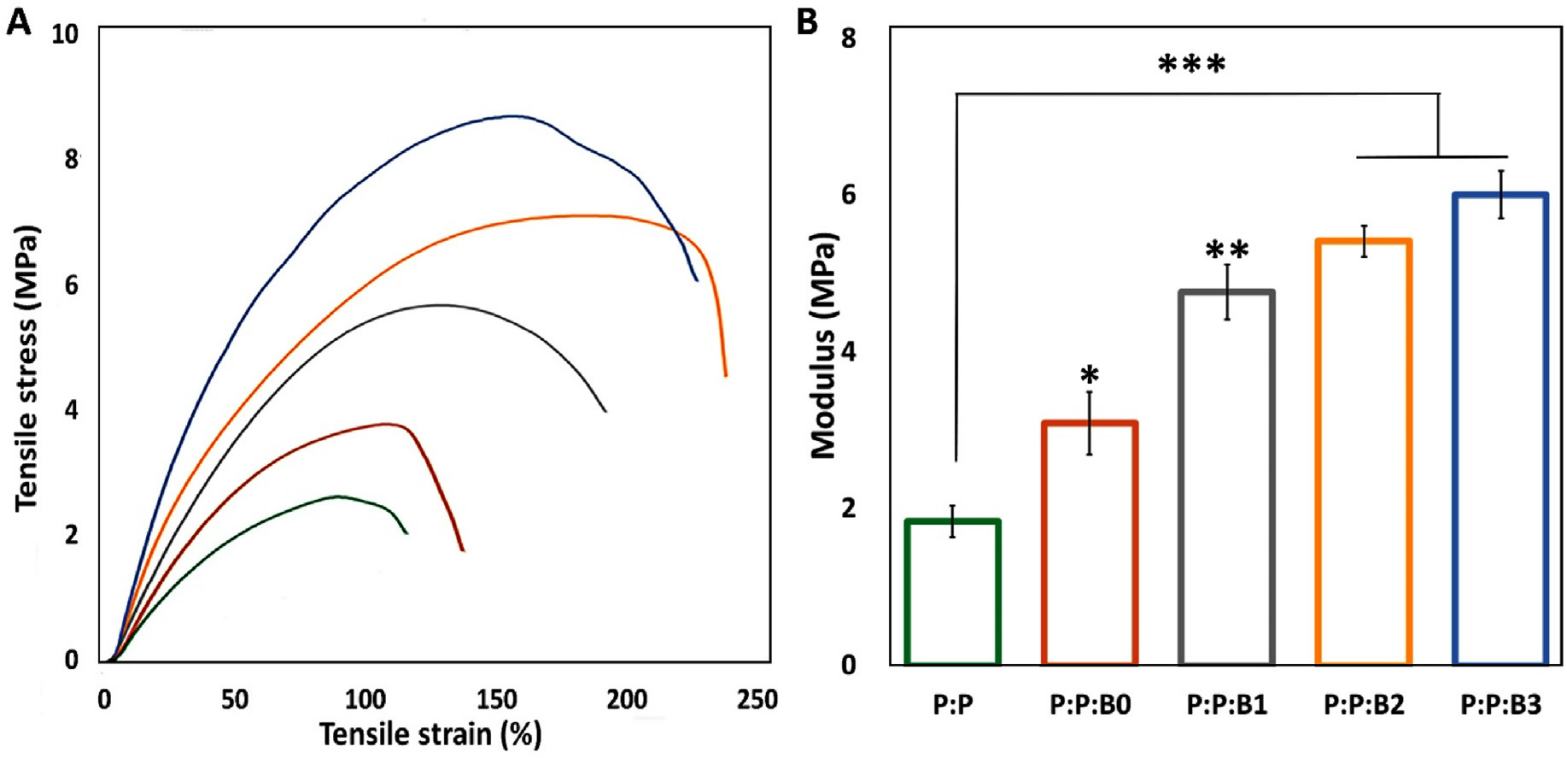
(A) Typical σ−ε curves of P:P and P:P:Bs grafts. (B) Young's modulus of vascular grafts. Note: Data presented as mean ​± ​SD, n ​= ​3, all statistical significance shown in comparison to control graft (P:P) unless otherwise stated, ∗p ​< ​0.05, ∗∗p ​< ​0.01, ∗∗∗p ​< ​0.001.

Static tensile measurements were employed to evaluate the mechanical properties of synthesized P:P and P:P:Bs vascular grafts (Fig. 5). The stress-strain curves of all vascular grafts showed a similar tendency, indicating that they were in an elastomeric condition. In addition, Young's modulus (E) and ultimate tensile strength (UTS) of the synthesized vascular grafts improved as the quantity of Cu supplemented to polymeric matrix increased, while for P:P:B3 graft, the strain at break (Ɛ) decreased, as summarized in Table 3.

**Table 3:** Mechanical properties and cross-linking density of P:P and P:P:Bs grafts.

Vascular grafts	Ɛ (%)	UTS (MPa)	Suture strength (N)	n (mol/m^3^)
**P:P**	125 ​± ​7	1.5 ​± ​0.3	1.9 ​± ​0.2	26.4 ​± ​5.4
**P:P:B0**	157 ​± ​11	2.6 ​± ​0.6	2.8 ​± ​0.3	66.3 ​± ​8.3
**P:P:B1**	212 ​± ​10	5.5 ​± ​0.9	3.7 ​± ​0.5	83.1 ​± ​6.7
**P:P:B2**	249 ​± ​17	7.1 ​± ​0.8	4.1 ​± ​0.3	99.8 ​± ​10.6
**P:P:B3**	231 ​± ​8	8.5 ​± ​0.4	5.2 ​± ​0.5	107.8 ​± ​7.2

E − Mechanical Young Modulus. UTS- ultimate stress strength. Ɛ-strain at break.

P:P graft presents a Young's modulus of 1.84 ​± ​0.16 ​MPa. Young's modulus of P:P:B3 graft had a seven-fold increase compared to the P:P graft and it may be caused by strong interactions between hydroxyl groups of the PGS polymer chains and ions in the structure of the modified BGs, especially Cu (Fig. 2 C and D). During mechanical solicitation, these interactions restrict the movement of polymer chains. As a consequence, this means that the P:P:Bs grafts will have a higher UTS because of the enhanced load transfer between the modified BGs and polymer chains. Due to the strong connection between the modified BGs and the matrix, especially when a high proportion of Cu-containing the modified BGs is introduced to the polymeric matrix, the mobility of the polymer chain is lowered, hence reducing the deformation at the break. As indicated previously, another component that contributes to this behavior is glutaraldehyde, which enhances the consumption of the hydroxyl groups of the PGS polymer chains, causing PGS cross-linking. When the modified BGs were added to vascular grafts, the elastic modulus values were in the same range as natural blood vessels, which is the gold standard for bypass surgery (2.5–8 ​MPa for radial and internal mammary arteries [46], and 4.2 ​MPa for saphenous veins [47]). Furthermore, vascular grafts incorporating the modified BGs had UTS values similar to native blood vessels, ranging from 0.5 to 3 ​MPa for coronary arteries, 1.8 ​MPa for saphenous veins [47], and 1.5–4.1 ​MPa for radial and mammary arteries [46]. Ɛ values for vascular grafts incorporating the modified BGs were higher than these natural vessels that have ranging from 60 to 250% [46]. In addition, the suture retention strength is an extremely significant consideration during the operation that is performed to implant the graft. Table 3 summarizes the suture retention strength of synthesized vascular grafts. The suture retention strength of vascular grafts incorporating modified BGs was found to be greater than that of human saphenous vein (1.8 ​N) and human mammary artery (1.4 ​N) [48]. Also, P:P:Bs vascular grafts had a suture retention strength that was greater than the minimum required for implantation (more than 2.0 ​N) [48]. The findings show that the P:P:Bs vascular grafts can offer suitable support for cell organization and vascular tissue formation while also providing enough strength to overcome circulatory pressure *in vivo.*

Eq. (S4) may be used to calculate the cross-linking density (n) of synthesized vascular grafts. Table 3 presents the cross-linking density evaluations with the modified BGs added to the polymeric matrix. The increase in cross-linking density attributed to the presence of the modified BGs, which limits polymer chain movement under uniaxial mechanical stress, is most likely responsible for the improved mechanical properties (Fig. 5). Due to the covalent bonds formed between the filler and the backbone of the polymer chains, Liang et al. discovered that incorporating micro-sized modified BGs (0–15% w/w) in PGS matrix can increase the elongation at break from 160 to 550% while increasing the Young's modulus of the composites by up to a factor of four [30].

The cross-linking density increased by more than four-fold, with values of 26.4 ​± ​5.4 and 107.8 ​± ​7.2 ​mol/m^3^ calculated for the P:P and P:P:B3 vascular grafts, respectively, suggesting that the modified BGs, in fact, ions released from the modified BGs, particularly Cu, contribute not only to physical entanglement but also to chemical cross-linking of the polymeric chains. The cross-linking density has a significant impact on the mechanical properties of elastomers [31]. This finding demonstrates that by employing an active filler, such as the modified BGs, that may generate new sites for polymeric chains to bind and enhance the cross-linking density, it is possible to improve the mechanical properties of grafts.

### 3.7 Swelling behavior and cross-linking density

Swelling experiments can also be performed to evaluate an elastomeric cross-linking density of materials. Using Eq. (S1), the solvent absorption capacity of the sample was estimated, and the results are presented in Table 4.

**Table 4:** Cross-linking density of P:P and P:P:Bs grafts using the swelling method.

Vascular grafts	Swelling percentage (%)	n (mol/m^3^, χ ​= ​0.48)
**P:P**	702.2 ​± ​32.4	31.1 ​± ​4.3
**P:P:B0**	533.3 ​± ​25.6	71.1 ​± ​5.2
**P:P:B1**	466.7 ​± ​17.5	94.2 ​± ​6.1
**P:P:B2**	422.2 ​± ​28.2	115.7 ​± ​8.5
**P:P:B3**	391.1 ​± ​15.8	130.3 ​± ​7.9

n – Cross-linking density. χ – Flory-Huggins polymer-solvent interaction parameter.

The swelling degree can be employed for an elastomeric matrix to calculate the cross-linking density, qualitatively and quantitatively. Using Eq. (S1), the calculated swelling degree demonstrates that with an increase in the amount of Cu doped into the modified BGs added to the polymer network, solvent uptake will decrease (Table 4). This shows that, by providing additional cross-linking sites for PGS chains to join, the modified BGs contribute to the formation of a polymer network, which is concurrent with mechanical properties (Fig. 5) and FTIR spectroscopy (Fig. 2).

The Flory-Huggins polymer-solvent interaction parameter remains constant for a particular solvent-polymer combination and is unaffected by the cross-linking density of the polymer chain [49]. Eq. (S3) was used to calculate the value of χ (based on swelling and modulus data for P:P graft), and a value of 0.48 was discovered. When the modified BGs and glutaraldehyde were supplemented to the polymer matrix, cross-linking density and mechanical theory, calculated using Eq. (S3) and Eq. (S4), showed a difference. This difference rises with an increase in the amount of Cu in the modified BGs added to the elastomeric matrix (Table 3). Based on cross-linking density, evaluated by swelling experiments, the modified BGs and glutaraldehyde are covalently bonded to the PGS polymer chains, increasing its cross-linking density, which confirms that for a particular pair of polymer-solvent, solvent interaction parameter is constant, while the only altered experimental parameter was the amount of Cu in the modified BGs. Also, in agreement with previous studies where fillers were added to PGS, the variances between the cross-linking densities calculated using the Flory mathematical model and the rubber elasticity theory suggest that incorporating BGs encourages physical entanglement of the macromolecular chains and also increases chemical cross-linking of the polymer [31,32].

### 3.8 Cell behavior on fabricated vascular grafts

#### 3.8.1 Proliferation and cytotoxicity assays

**Fig. 6:**
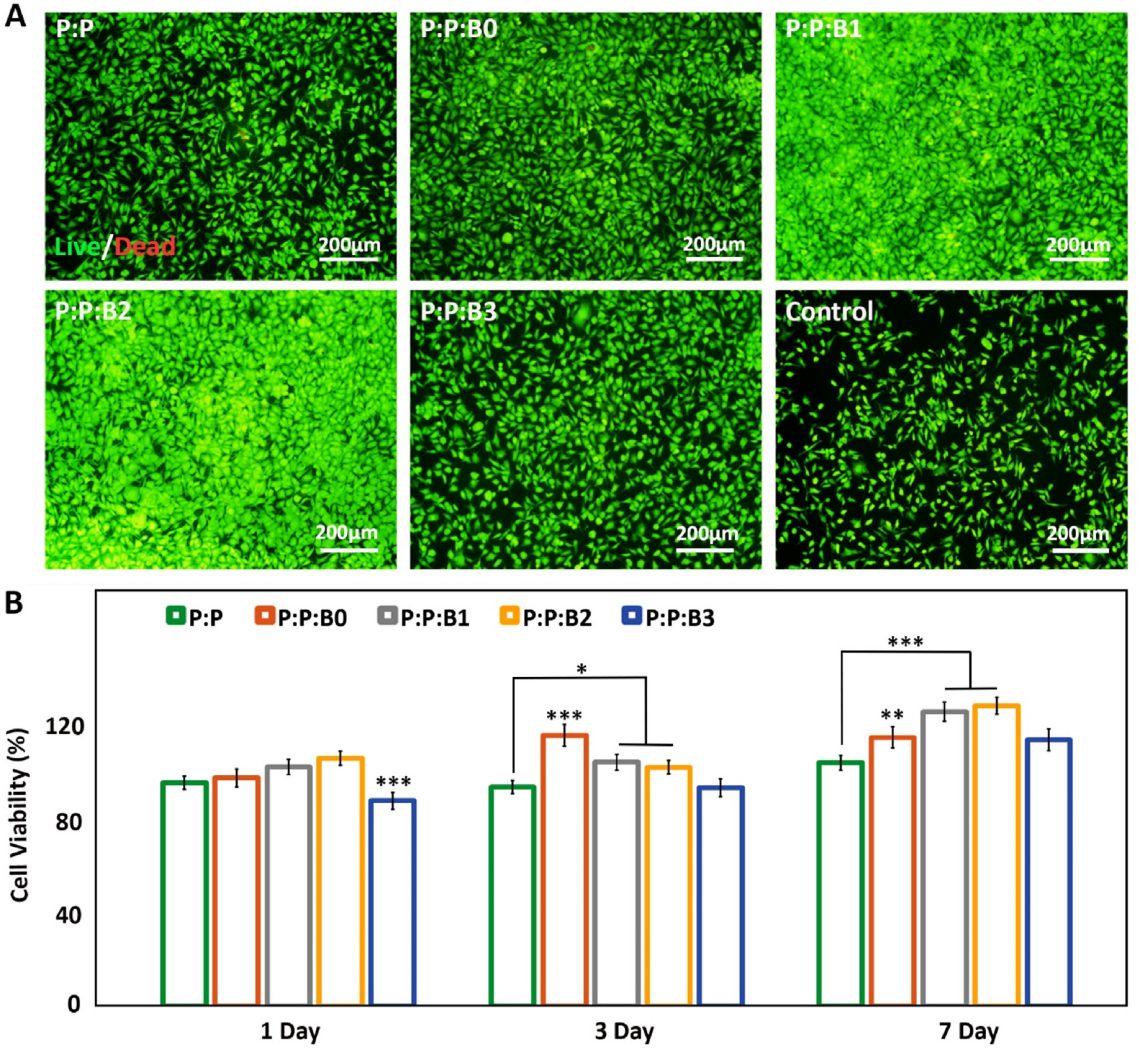
(A) HUVECs proliferation and morphology after 1 day of incubation with leachate the vascular grafts and control group obtained by fluorescence microscopy, indicating Cu-releasing vascular grafts stimulated proliferation of HUVECs (scale bar: 200 ​μm). (B) Vascular grafts' cytocompatibility evaluation. MTT assay measured EC viability after incubation for 1, 3, and 7 days with P:P:Bs vascular grafts and control graft. Data presented as mean ​± ​SD, n ​= ​3, all statistical significance shown in comparison to P:P graft, ∗p ​< ​0.05, ∗∗p ​< ​0.01, ∗∗∗p ​< ​0.001.

The clinical effectiveness of synthetic vascular grafts depends critically on endothelium regeneration of the luminal surface. Following the implantation of synthetic vascular grafts, ECs move from the neighboring vessel and proliferates from cut borders of the lumen toward the center of the lumen to produce a full monolayer [50]. Mobility and migration of ECs expansion throughout the endothelialization process would be crucial for tissue regeneration. ECs proliferation stimulation has been demonstrated to be one of the physiological functions of BGs, particularly Cu-doped BGs [51]. In order to examine the proliferation and morphology of HUVECs after 24 ​h, fluorescent microscopy was employed as a proof of concept. After 24 ​h of incubation with various leachate media, the fluorescent images of HUVECs are shown in Fig. 6A. The presence of the modified BGs increased cell proliferation, which was consistent with the findings of other investigations [51,52]. According to Fig. 6A, the Cu-releasing vascular grafts, P:P:B1 and P:P:B2 grafts, induced an increase in cell proliferation compared to control groups; however, an excess of Cu inhibited cell growth and proliferation in the P:P:B3 graft. In all the vascular grafts, cells have a dispersed morphology that shows an ideal environment for cell proliferation; but, when the Cu content of the vascular grafts is increased by more than 5 ​wt%, the cells tend to become less confluent. This cellular elongation is necessary for angiogenesis and the development of networks. According to the result, *in vivo* endothelial regeneration can be accelerated by the P:P:Bs vascular grafts by encouraging ECs proliferation.

It is vital to evaluate the cytotoxicity of new products because biomaterials and chemical agents employed in cell treatment may influence cell viability and metabolism. MTT test was used to evaluate the cytotoxicity of P:P:Bs and control graft. The results in Fig. 6B demonstrate the non-cytotoxic effects of produced grafts over seven days. This conclusion is consistent with other studies, which found that scaffolds containing BGs had no harmful effects [53,54]. Additionally, the results showed that, compared to the control groups, the EC proliferation rate was significantly higher after one day of incubation with P:P:B1 and P:P:B2 grafts. The proliferation of ECs was also much higher after 3 and 7 days than after one day, showing that the presence of synthetic vascular grafts supports cell proliferation. The higher rate of 10.13039/501100000780EC proliferation caused by the addition of Cu doped in composites and previous reports of biocompatibility are both supported by our findings. However, after 3 and 7 days, there was a statistically significant fall in the viability of HUVECs when the P:P:B3 graft was present compared to the control group. Reactive oxygen species (ROS) forming, is one of the primary mechanisms by which Cu induces cytotoxicity in cell monolayers *in vitro.* An increase in ROS generation at high Cu concentrations might potentially cause DNA damage and mutations due to interactions with nuclear chromatin and peroxidation of biomolecules, including lipids and proteins in cell membranes [55].

#### 3.8.2 Migration assay

**Fig. 7:**
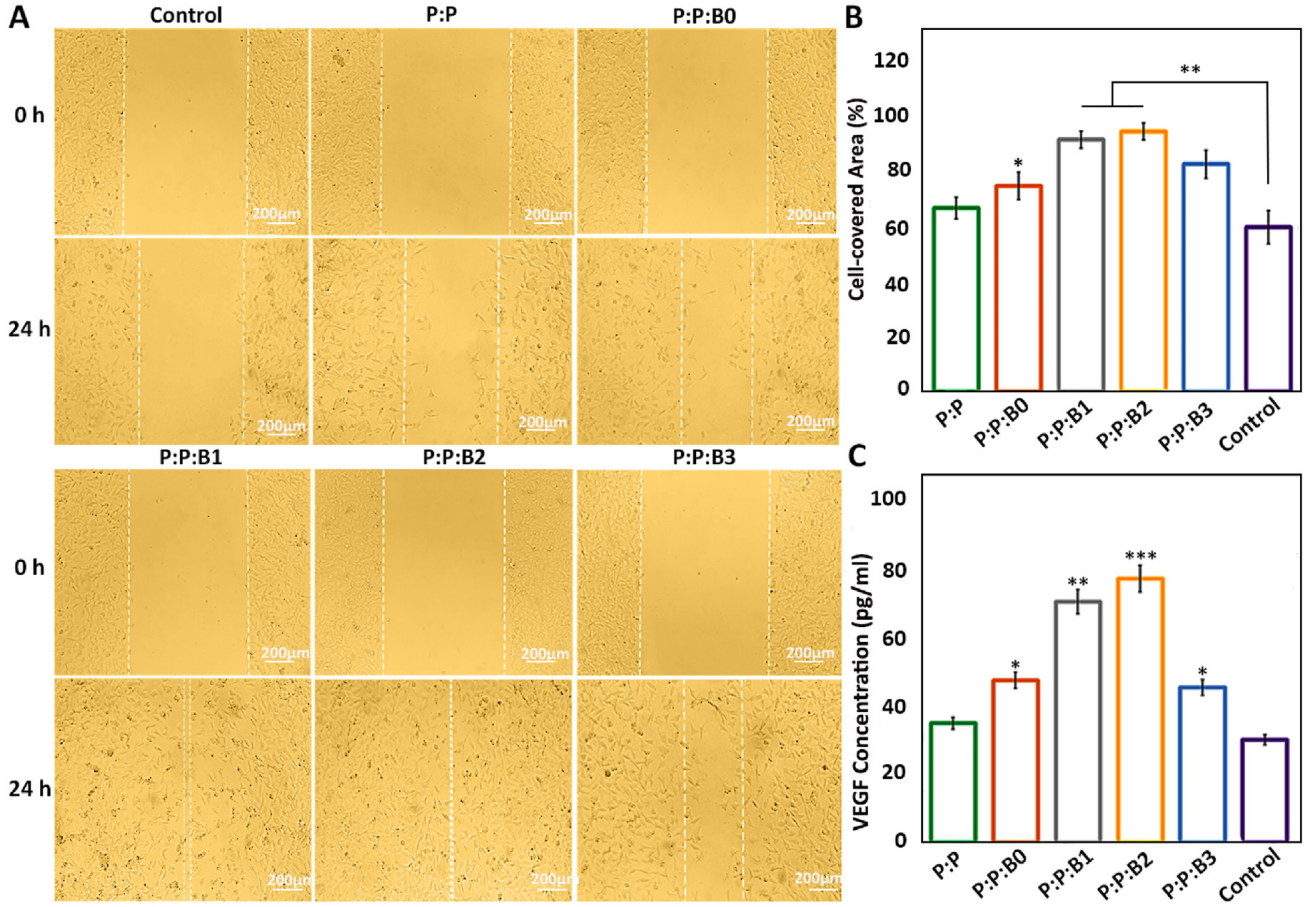
HUVECs Migration after 1 day of incubation with different culture mediums or leachates. (A) HUVECs monolayer was scratched using a tip of a 100 ​μl pipette. The position of the initial scratch edge is illustrated with dotted lines, at the beginning of the experiment and after a day of incubation with different culture mediums or leachates. (B) The ratio (%) of cell-coverd area was evaluated after 1 day compared to the initial gap between scratch borders. (C) Quantification of secreted VEGF from HUVEC for different groups on day 5. Note: scale bars 200 ​μm. Data presented as mean ​± ​SD, n ​= ​3, all statistical significance shown in comparison to control group unless otherwise stated, ∗p ​< ​0.05, ∗∗p ​< ​0.01, ∗∗∗p ​< ​0.001.

The lumen surface of vascular grafts must be covered with ECs after implantation. Migration of ECs toward the surgical site that was injured during implantation is an important aspect that might enhance the healing process following surgery [3,56]. As a result, thrombosis and bacterial infection risks may be decreased by a quicker regeneration of the endothelial monolayer after implantation. Fig. 7A demonstrates ECs migration after incubating for 1 day in presence of various leachates of the vascular grafts or culture medium. With assistance from the WimScratch web tool, the acquired photos were quantitatively evaluated. The results demonstrate that the migration rate of ECs was significantly enhanced in the modified BGs containing vascular grafts, and after a day, the scratched surface was covered by 75%, 92%, 95%, and 83% in P:P:B0, P:P:B1, P:P:B2 and P:P:B3, respectively (Fig. 7B). The fact that there is not much of a difference in the rate of cell migration between the control graft (P:P) and the positive control indicates that the ions in the modified BGs, particularly Cu and B, are responsible for the accelerated migration rate, which promises quicker cell covering after implantation. According to earlier research, B and Cu activated the mitogen-activated protein kinase (MAPK) signal pathway, which in turn caused HUVEC to proliferate and migrate [57,58]. By encouraging the release of the angiogenic marker VEGF in HUVECs, Cu ions were also proven to promote angiogenesis [59]. Our findings are consistent with earlier research on BGs that showed a notable improvement in HUVEC proliferation and migration in the culture medium [12,33]. The cell behavior evaluations suggest that the incorporation of modified BGs in vascular grafts accelerates endothelialization of the lumen surface. The endothelium layer acts as an anticoagulant and antimicrobial surface and has the potential to substantially improve implantation success rates.

#### 3.8.3 VEGF secretion

VEGF is an effective signaling protein that cells make to promote angiogenesis and vasculogenesis. When blood circulation is insufficient, as it is in hypoxic conditions, the functional role of VEGF is to restore the oxygen supply to ischemic tissues. Following treatment with a specific extraction medium, we measured the VEGF release from HUVECs. Fig. 7C demonstrates how all the P:P:Bs grafts stimulate HUVECs to secrete more VEGF into the culture medium in time. For 5 days, VEGF secretion from the P:P:B1 and the P:P:B2 grafts was more than twice as high as that from the control group and the P:P graft. These results demonstrated that Cu induces angiogenic factors and the differentiation of HUVEC into vascular ECs. Previous research has focused on the Cu-induced angiogenesis mechanism [60]. The hypoxia-inducible factor-1 (HIF-1) element, a transcription factor that regulates VEGF production, may be induced by both Cu and hypoxia. However, the excess Cu can prevent the HIF-1 transcriptional complex from forming, which in turn prevents the synthesis of VEGF [61]. The Cu concentration in living systems must be bioavailable for it to be useful. According to Fig. 7C, the excess Cu in the P:P:B3 graft caused to reduce in the production of VEGF from HUVECs into the culture medium, compared to other Cu-containing grafts. The ability of ECs to proliferate, migrate, and express VEGF is crucial for the angiogenesis process, which plays a crucial part in the process of defect regeneration [11].

Overall, the HUVECs' *in vitro* angiogenesis activity analysis indicated that the modified BGs incorporating vascular grafts stimulated higher levels of proliferation, migration, and VEGF protein expression from the HUVECs, particularly in the presence of the P:P:B2 graft, suggesting that it may be ideal for use as a synthetic vascular graft.

### 3.9 Antibacterial properties

Most contamination of prosthetic vascular grafts occurs in the peri-operative period or during graft transplantation [62]. Numerous research has focused on the antimicrobial effect of borate BGs because of the quick rate of dissolution and release of multifunctional ions *in vitro* and *in vivo* [37]. It has been confirmed that BGs have antibacterial effects against a variety of bacteria and fungi, as well as therapeutic properties. The osmotic pressure and pH variation caused by BGs dissolution are responsible for their antibacterial activity [63]. Small concentrations of BGs preserve their antibacterial effectiveness even after prolonged exposure, and bacteria are less likely to develop resistance mechanisms to their antibacterial properties compared to antibiotics [64]. In antibacterial evaluations, the release of B and Cu ions by the modified BGs in response to an acidic environment that may develop during the bacterial growth phase is responsible for the antibacterial properties of the synthesized vascular grafts. Two possible antibacterial pathways of B are the depletion of energy by binding to NAD and NADH and the initiation of DNA damage by binding to ribose groups [65]. Also, it has been demonstrated that oxidative damage to membrane phospholipids caused by Cu damages the integrity of bacterial membranes. In addition, Cu ions are capable of penetrating bacteria, which can lead to DNA damage and oxidative stress [66].

**Fig. 8:**
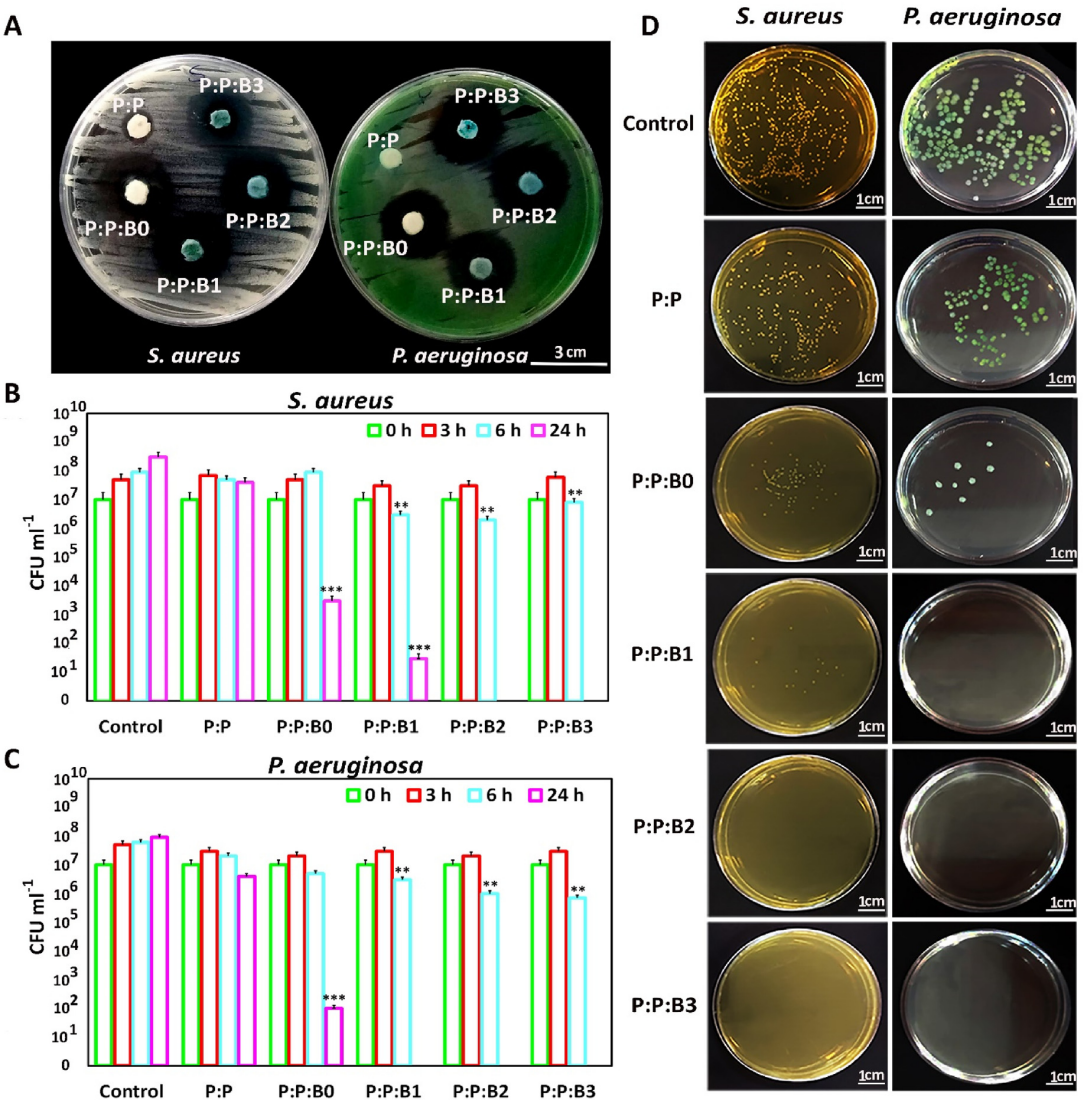
Antibacterial properties of P:P and P:P:Bs grafts. (A) Evaluation of the vascular grafts for Antibacterial Activity Using a Disc Diffusion Assay. Plate counting of the amount of viable (CFU mL^−1^) (B) *S. aureus* and (C) *P. aeruginosa* incubated with P:P and P:P:BS grafts. The data was gathered on days 0, 3, and 24 of incubation. (D) Agar plates after 24 ​h incubation of *S. aureus* and *P. aeruginosa* with P:P and P:P:Bs grafts. Data presented as mean ​± ​SD, n ​= ​3, all statistical significance shown in comparison to control group unless otherwise stated, ∗p ​< ​0.05, ∗∗p ​< ​0.01, ∗∗∗p ​< ​0.001.

In this study, the inhibitory zone experiment was used with Gram-positive and Gram-negative strains to examine the antibacterial activity of the synthesized vascular grafts. Bacterial growth on the surface of the biomaterial is anticipated to be inhibited by the sustained ions release. In this method, the antibacterial effects of biomaterials are directly linked to the prevention of bacterial growth. Fig. 8A and Fig. S3, in supporting information, show the inhibitory halos of the P:P:Bs vascular grafts and the control graft on agar plates after 24 ​h at 37 ​°C. The P:P:Bs vascular grafts reduced bacterial growth in both strains with a zone of inhibition that was about 25 ​mm in diameter.

Additionally, the CFU counting method was utilized to examine the antibacterial activity of P:P:Bs grafts and control grafts. For this method, all grafts were incubated with 10^7^ ​CFU/ml *S. aureus* and *P. aeruginosa* under static conditions. The CFU count at various time intervals was displayed in Fig. 8 B, C and D. After 24 ​h, the CFU counting method revealed a 99.99–100% reduction in microorganisms in the presence of P:P:Bs grafts. However, bacteria cultured with control grafts continued to grow and showed a constant level that was congruent with the result of inhibition zone halos. In the antibacterial evaluation of vascular grafts containing modified BGs, the inhibitory haloes were more than twice as wide as those observed in borate-based BGs in previous studies [67], indicating a significant improvement in the antibacterial properties of synthesized BGs with the modified composition. These observations provide confirmation for the assumption that vascular grafts containing modified BGs can significantly reduce bacterial viability following implantation.

### 3.10 Hemocompatibility

**Fig. 9:**
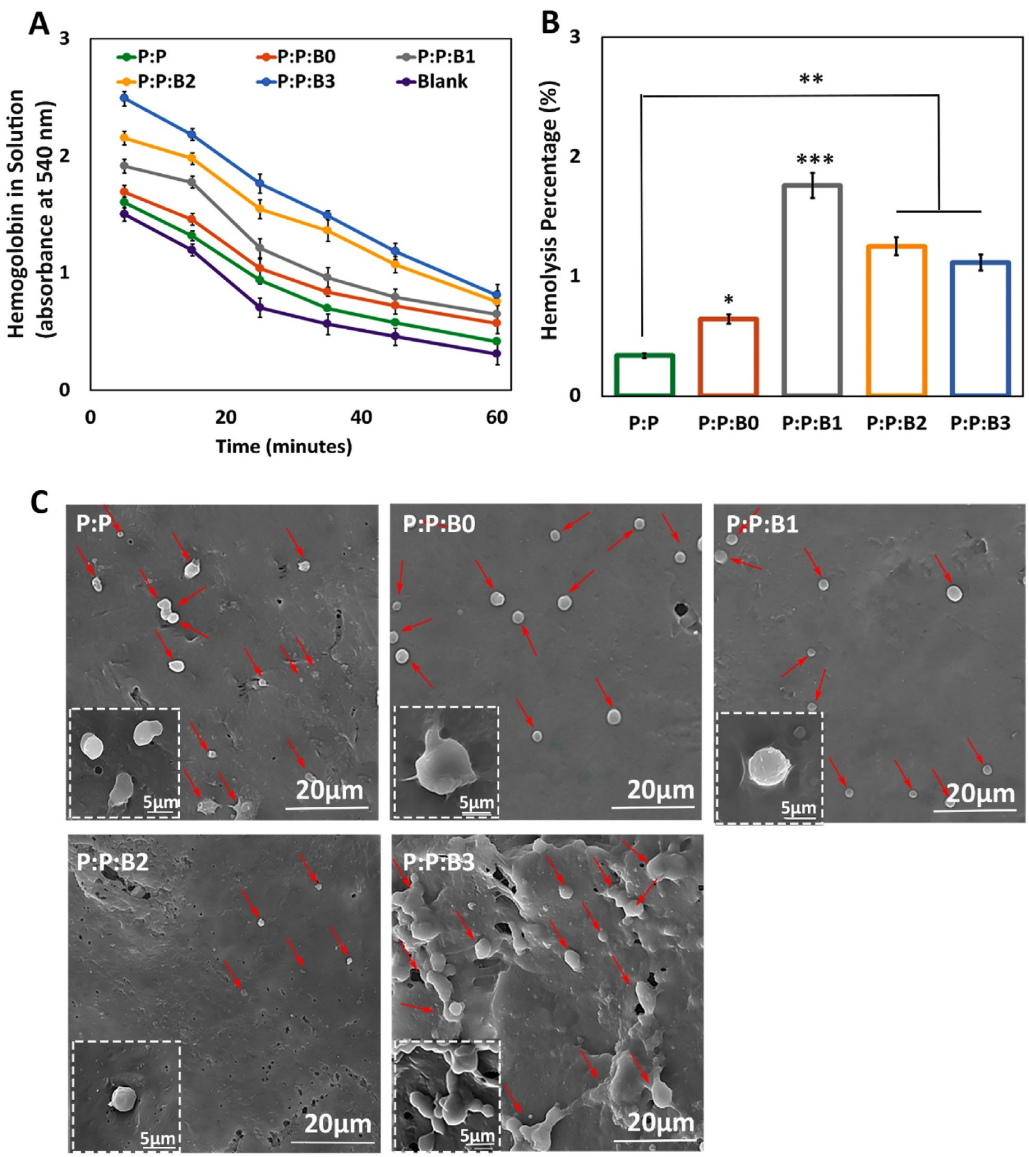
(A) Blood clotting profile and; (B) hemolysis percentage of lumen surface of P:P and P:P:Bs vascular grafts. (C) Platelets that have attached to grafts (shown by the red arrows) after being incubated with fresh human PRP for 1 ​h with the different groups of vascular grafts. Scale bar ​= ​20 and 5 ​μm. Data presented as mean ​± ​SD, n ​= ​3, all statistical significance shown in comparison to control graft (P:P) unless otherwise stated, ∗p ​< ​0.05, ∗∗p ​< ​0.01, ∗∗∗p ​< ​0.001. (For interpretation of the references to colour in this figure legend, the reader is referred to the Web version of this article.)

Several physiological responses are possible when blood contacts a biomaterial surface, such as thrombus formation and RBC rupture. As a result, while dealing with vascular prostheses, hemocompatibility is one of the most crucial factors to consider [29]. In this study, kinetic clotting time technique, hemolysis, and platelet adhesion were used to assess the hemocompatibility of the lumen surface of the synthesized vascular grafts. Fig. 9A depicts the blood clotting behavior in the presence of vascular grafts. For the purpose of determining how the materials influence clotting time, an *in vitro* static clotting time investigation is carried out. Free hemoglobin absorbs variably over time in a solution; the higher absorbance, the better anti-thrombotic capabilities. Fig. 9A demonstrates that blood incubated with the P:P and the P:P:Bs grafts had a much greater absorbance than the control group at almost every predetermined time point. After 30 ​min, the P:P:B3 and P:P:B2 vascular grafts exhibit the most potent anticoagulant effects. As can be observed, Cu-containing grafts are more effective than P:P grafts. This finding suggested that the Cu ion in the P:P:Bs vascular grafts may effectively suppress the activation of the intrinsic blood coagulation system, which is in congruence with previous studies [68]. The presence of a –COOH functional group on the surface of the biomaterial may also affect its hemocompatibility. To this end, Sperling et al. revealed that the amount of –COOH functional groups on the surface of a substance stimulates the activation of coagulation [69]. The FTIR analysis showed that the P:P vascular graft had the highest concentration of surface –COOH functional group, indicating that it activated coagulation more than the other grafts. As a result, the incorporation of modified BGs into vascular grafts led to a reduction in the number of –COOH functional groups, which is associated with decreased coagulation. It is also important to note that BGs composition was modified by removing coagulant elements; this meant that including them in vascular graft composition didn't result in clot formation, confirming that the modification was effective.

Hemolysis is a destructive phenomenon characterized by the destruction of RBC in the presence of blood abnormalities. These abnormalities may be driven by, for example, the introduction of a toxic foreign element into the blood [70]. Thus, before clinical uses, it is critical to assess a material compatibility test with blood cells. Fig. 9B demonstrates that the hemolysis percentage increases with the addition of the modified BGs to the P:P matrix. According to previous research, hemolysis is caused by an excess of Cu in several clinical conditions [71]. High Cu doses rapidly and considerably reduce the deformability of erythrocytes. Because this decrease in cell flexibility happens simultaneously with a significant increase in membrane permeability and osmotic fragility in Cu-exposed cells, it is likely that changes to the erythrocyte membrane caused by Cu are responsible for the decrease in erythrocyte survival [71]. The higher level of Cu released in the first half hour is considered to be the cause of an increase in the hemolysis rate of P:P:B1 graft when compared to other grafts. The data shows that all the synthesized vascular grafts with or without the modified BGs are very compatible with human blood. According to the ISO 10993-4 standard, the acceptable hemolytic level for biomaterials is 5% [29]; nevertheless, our data showed <1.7% lysis for all the grafts, indicating that bilayer vascular grafts, containing the modified BGs, had no erythrocyte destruction.

The main criteria for hemocompatibility to evaluate the blood-contacting biomaterials are platelet adhesion and activation. After platelets were incubated with vascular grafts for 1 ​h, their morphology was analyzed by SEM imaging. Platelets adhering to a control graft showed activated morphology, with an irregular shape and the formation of pseudopods, whereas platelets adhering to P:P:B0, P:P:B1, and P:P:B2 grafts showed a discoid and non-activated morphology (Fig. 9C). The lower platelet adhesion observed in the P:P:B1 and the P:P:B2 grafts may be due to the presence of the Cu atom, which helps to suppress platelet adhesion that is in line with the previous study [72]. The morphology of the platelets on the P:P:B3 vascular graft, however, revealed that platelet activation had occurred. Since glutathione (GSH) is present in platelets, it is possible that the activation of platelets and the subsequent formation and spread of pseudopodia on the P:P:B3 graft might be attributed to the potential of excess Cu to produce ROS via the Fenton reaction and to bind GSH [73]. In conclusion, the hemocompatibility data indicated that BGs might be successfully employed in blood-contact applications by modifying their composition and using their specific features, such as angiogenesis and antibacterial, without causing coagulation problems.

### 3.11 In vivo angiogenesis and gene expression analysis

**Fig. 10:**
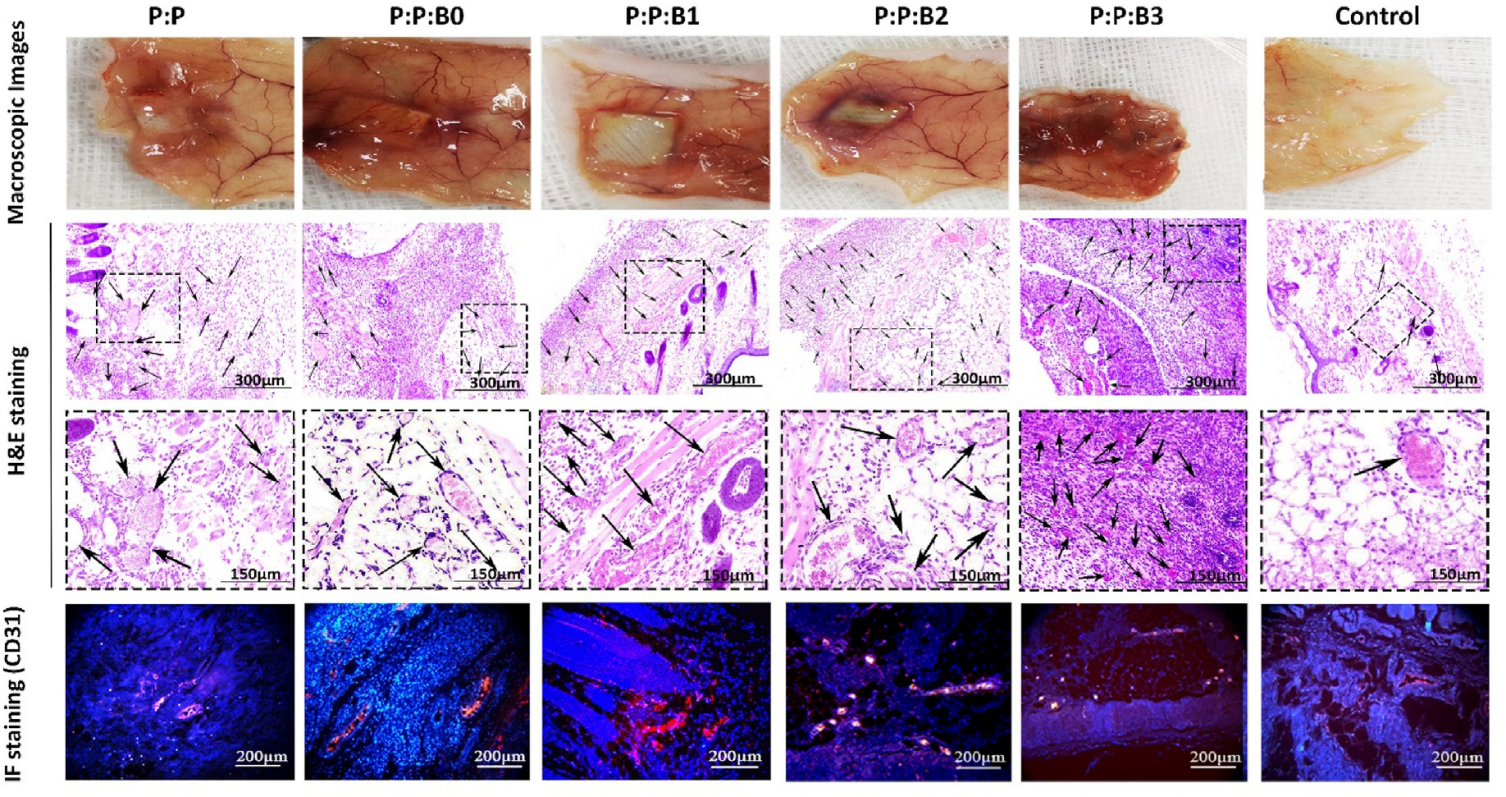
Macroscopic observation, Histological staining (H&E) and Immunofluorescence staining (CD31) of subcutaneously implanted P:P and P:P:Bs grafts. H&E and CD31 staining showing accelerated angiogenesis (arrows) in P:P:B1 and P:P:B2 grafts. Despite high angiogenesis in P:P:B3 graft, the inflammatory response was observed.

**Fig. 11:**
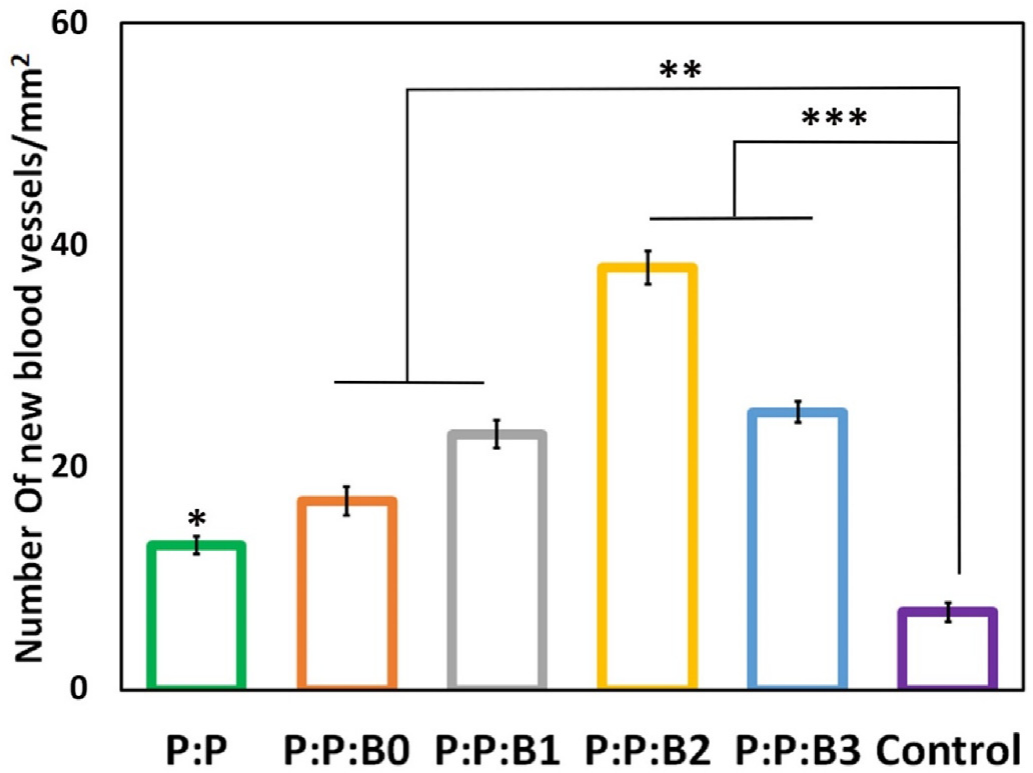
Quantitative analysis of the formation of new blood vessels. Data presented as mean ​± ​SD, n ​= ​3, all statistical significance shown in comparison to control group unless otherwise stated, ∗p ​< ​0.05, ∗∗p ​< ​0.01, ∗∗∗p ​< ​0.001.

Macroscopic images of the subcutaneous implants showed that the P:P:Bs vascular grafts caused the formation of more blood vessels than the P:P graft and control group (Fig. 10). This conclusion was supported by H&E staining, demonstrating that the P:P:Bs grafts' implantation accelerated vascularization. Similar to the *in vitro* experiments, both P:P:B1 and P:P:B2 vascular grafts show significantly higher levels of angiogenesis compared to the control graft. Additionally, all vascular grafts were found to be biocompatible, except for the P:P:B3 graft, with no adverse reactions or inflammation. This is promising because it demonstrates that while the antimicrobial materials successfully remove bacteria, they have no adverse effects on nearby healthy tissue. The inflammatory response that appears after 8 days may be caused by surgical trauma during the vascular grafts' insertion into the pouch as well as by the potentially toxic consequences of the extra Cu in the P:P:Bs grafts. The P:P:B3 graft had a substantially stronger inflammatory response than the others, which is consistent with MTT data and may be due to the excess Cu in this graft. The CD31 immunofluorescent staining demonstrated that the modified BG-containing vascular grafts increased the number of new blood vessels 4 weeks after implantation (Fig. 10). The quantitative analysis of the formation of new blood vessels revealed the number of vessels in the P:P:B2 graft was substantially higher than in the other vascular grafts (Fig. 11), corresponding with the *in vitro* evaluation results. It has been reported that higher levels of CD31 expression play a role in the process of reendothelialization. Previous research confirmed the stimulating effect of Cu ions on reendothelialization *in vivo* by showing that immunofluorescence staining for CD31 increased significantly in Cu-doped BGs scaffolds [7]. The incorporation of biomolecules (such as VEGF and bFGF) onto the surface of vascular graft materials is an intriguing area of research in vascular tissue engineering because it improves the chances of successful reendothelialization; however, this approach is limited by high production costs, an unclear result for surrounding tissues, and the risk of negative biological effects [7,74]. In this study, Cu-doped modified BGs were used in the inner layer of vascular grafts to increase reendothelialization and angiogenesis. These modified BGs have a low cost, high stability, and maybe improved clinical safety [7], indicating that this technique may efficiently enhance ECs recruitment association with angiogenesis enhancement.

**Fig. 12:**
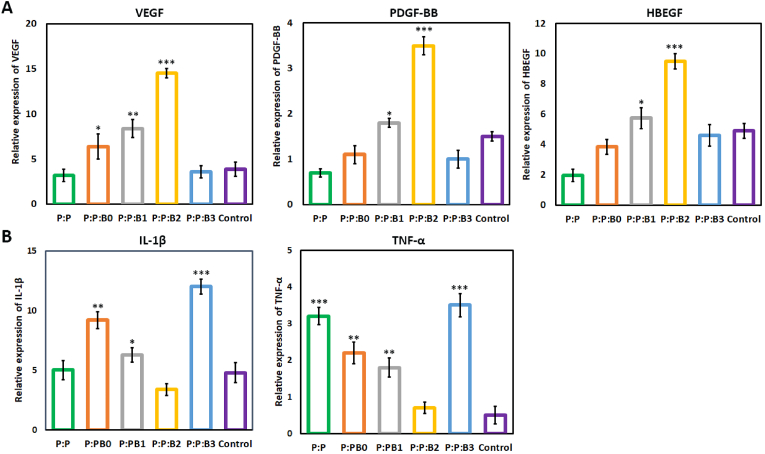
Gene expression of different vascular grafts. (A) Gene expression of proteins typically associated with the initiation of angiogenesis (VEGF), the maturation of growing blood vessels (PDGF-BB, HBEGF), and (B) proteins involved in inflammation (TNF-α, IL-1β). Data presented as mean ​± ​SD, n ​= ​3, all statistical significance shown in comparison to control group unless otherwise stated, ∗p ​< ​0.05, ∗∗p ​< ​0.01, ∗∗∗p ​< ​0.001.

Gene expression analysis of different vascular grafts was shown in Fig. 12 and revealed that P:P:B2 graft strongly downregulated the inflammatory proteins IL-1β and TNF-α. These levels were lower but not statistically different from the control group. It was demonstrated that some ions in BGs, such as B, Mg, and Cu, can reduce the inflammatory response in macrophages as indicated by the downregulated expression of pro-inflammatory cytokines (e.g., TNF-α, IL-1β, and IL-6) [75]. However, excess copper levels induce oxidative stress responses that activate inflammatory responses [61], which confirms the results related to the P:P:B3 vascular grafts. High quantities of platelet-derived growth factor-BB (PDGF-BB) were also expressed by the P:P:B2 graft. PDGF-BB is well-known to recruit pericytes and mesenchymal stem cells, which then stabilize the developing vasculature. Without this activity, the blood vessels that have been stimulated by VEGF will be in an immature state, prone to regression, and likely to leak [76]. High levels of heparin-binding EGF-like growth factor (HBEGF) expression in the P:P:B2 graft demonstrate that it can also recruit pericytes [77]. In contrast to blood vessels created by VEGF alone, those established by co-expressing VEGF and PDGF-BB lasted for a longer amount of time due to paracrine signals from recruited myeloid cells that did not require the involvement of pericytes [78]. So, for a high level of cell sheet survival and to make richly vascularized tissue, angiogenic factors need to be expressed together. Previous studies by Zhang et al. [79] showed the great advantage to blood vessel regeneration offered by the both delivery of VEGF and PDGF using electrospun vascular grafts, which accelerated ECs growth in the first 6 days and regulated slow smooth muscle cells (SMCs) growth in the first 3 days while providing accelerated growth after day 6. Even after four weeks of *in vivo* transplantation of rabbit carotid artery, no thrombosis or rupture was observed, and both ECs and SMCs proliferated on the inside and outside of vascular grafts, respectively. This study demonstrates the potential for the electrospun vascular graft to enhance revascularization by simultaneously delivering VEGF and PDGF [79]. Also, as mentioned above, the literature suggests that Cu influences multiple angiogenesis-related proteins, all of which play important roles in the process's commencement, maturation, and control of blood vessel formation. Two different signaling pathways play a significant role in copper's induction of angiogenesis at the molecular level. A significant one of these routes involves cu-activated HIF-1 and its role in angiogenic activation. Furthermore, the MAPK signaling pathway, which has key functions in both cell growth and angiogenesis, has been linked to Cu-induced angiogenesis [8]. The current study also showed that the P:P:B1 and P:P:B2 vascular grafts (with 3 and 5 ​wt% Cu, respectively) caused the highest expression of genes related to angiogenesis (VEGF, PDGF-BB, and HBEGF) compared to the control group. ECs play a crucial part in angiogenesis by locally proliferating in response to angiogenic stimulation [80]. The *in vivo* angiogenic potential of the P:P:Bs grafts is consistent with our *in vitro* angiogenesis (HUVECs proliferation, migration and VEGF secretion). The results of the current study support the angiogenic potential of the modified BGs, and it can be inferred that by employing modified BGs-loaded vascular grafts, the coverage of ECs in their lumen might be enhanced. The results of this investigation are consistent with a prior study that examined the angiogenic potential of Cu-doped BGs [7,11,52]. The *in vitro* and *in vivo* stimulation of angiogenesis in response to the modified BGs, particularly those that contain Cu, can prevent the synthesized vascular grafts infection [81]. In addition, this angiogenesis activity, which is necessary for the repair of tissues, demonstrates a great deal of promise for the repair and integration of tissues that happens after implantation [6].

## 4 Conclusion

In the presented study, bilayer vascular grafts with small diameters were fabricated through a combined fabrication strategy using 3D printing and electrospinning techniques as a possible substitute for vessel transplantation. Also, in this study, BGs with a modified composition were synthesized and incorporated in the inner layer to accelerate regeneration of the lumen surface of bilayer vascular grafts. The mechanical properties of porous, degradable vascular grafts containing the modified BGs were comparable to those of natural human arteries. All fabricated vascular grafts showed high biocompatibility. Incorporating modified BGs into vascular grafts improved rapid endothelialization via increased ECs' proliferation, migration, and VEGF secretion, which can accelerate tissue regeneration in the implanted site by preventing thrombosis and infection. The removal of hemolytic elements from BGs caused the synthesized vascular grafts, especially P:P:B1 and P:P:B2 grafts, to demonstrate appropriate anticoagulation properties with negligible hemolysis percentages and platelet activation; however, excess Cu in P:P:B3 grafts stimulated platelet activation. The Cu-releasing vascular grafts exhibited significant antibacterial effects against both gram-positive and gram-negative bacteria. According to *in vivo* angiogenesis analysis, H&E and CD31 immunofluorescent staining, all the vascular grafts containing modified BGs showed angiogenesis properties and increased new blood formation after implantation. Additionally, angiogenic growth factors were significantly up-regulated in P:P:B2 vascular grafts, while inflammatory proteins were significantly down-regulated, according to *in vivo* gene expression data. In light of these promising results, the incorporation of appropriately modified BGs into vascular graft prostheses may offer a promising approach for developing multifunctional constructs with accelerated regeneration and bactericidal properties without the risk of coagulation.

## Credit author statement

**Neda Alasvand**: Writing – original draft, Methodology, Validation, Investigation; **Aliasghar Behnamghader**Validation, Investigation, Resources, Supervision; **Peiman B. Milan**: Methodology, Validation, Resources, Supervision, Funding acquisition; **Sara Simorgh**: Writing – original draft, Methodology, Investigation; **Ali Mobasheri**: Writing – review & editing, Supervision; **Masoud Mozafari**: Conceptualization, Writing – review & editing, Supervision, Project administration, Funding acquisition

## Ethical approval

IR.IUMS.REC.1401.806.

## Declaration of competing interest

The authors declare that they have no known competing financial interests or personal relationships that could have appeared to influence the work reported in this paper.

## Data Availability

Data will be made available on request.
