# Supplementary material for: Tissue-engineered small-diameter vascular grafts containing novel copper-doped bioactive glass biomaterials to promote angiogenic activity and endothelial regeneration
Source: Mater Today Bio. 2023 Apr 27;20:100647. doi: 10.1016/j.mtbio.2023.100647 (PMC10232732; doi:10.1016/j.mtbio.2023.100647)
Supplement: Multimedia component 1 [file mmc1.docx]

**SUPPORTING INFORMATION**

**Tissue-engineered small-diameter vascular grafts containing novel copper-doped bioactive glass biomaterials to promote angiogenic activity and endothelial regeneration**

Neda Alasvand ^1^, Aliasghar Behnamghader ^1^, Peiman Brouki Milan ^2,3^, Sara Simorgh ^2,3^, Ali Mobasheri ^4,5,6,7^, Masoud Mozafari ^4,*^

^1^ Bioengineering Research Group, Department of Nanotechnology and Advanced Materials, Materials and Energy Research Center (MERC), Tehran, Iran

^2^ Department of Tissue Engineering & Regenerative Medicine, Faculty of Advanced Technologies in Medicine, Iran University of Medical Sciences, Tehran, Iran

^3^ Cellular and Molecular Research Center, Iran University of Medical Sciences, Tehran, Iran

^4^ Research Unit of Health Sciences and Technology, Faculty of Medicine, University of Oulu, Oulu, Finland

^5^ Department of Regenerative Medicine, State Research Institute Centre for Innovative Medicine, Vilnius, Lithuania

^6^ Department of Joint Surgery, First Affiliated Hospital of Sun Yat-sen University, Guangzhou, China

^7^ World Health Organization Collaborating Centre for Public Health Aspects of Musculoskeletal Health and Aging, Liege, Belgium

***Correspondence to:**

M. Mozafari, Ph.D; Email: mozafari.masoud@gmail.com; masoud.mozafari@oulu.fi

P. Brouki Milan, PhD; Email: peiman.brouki@gmail.com; brouki.p@iums.ac.ir


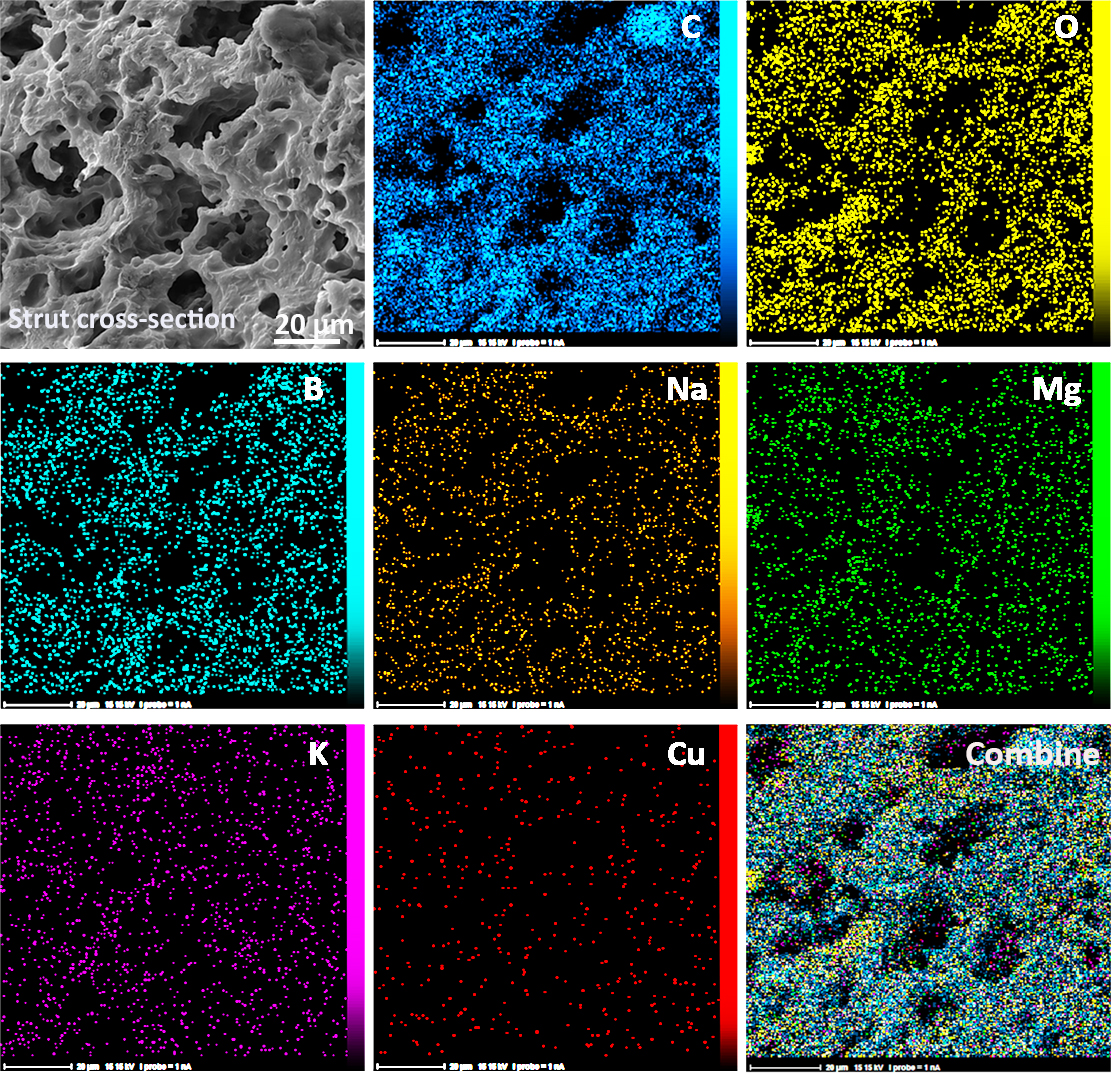


***Figure S1.*** *EDS mapping analysis of the P:P:B2 graft strut cross-section showing the uniform distribution of the modified BGs elements in the polymeric matrix.*


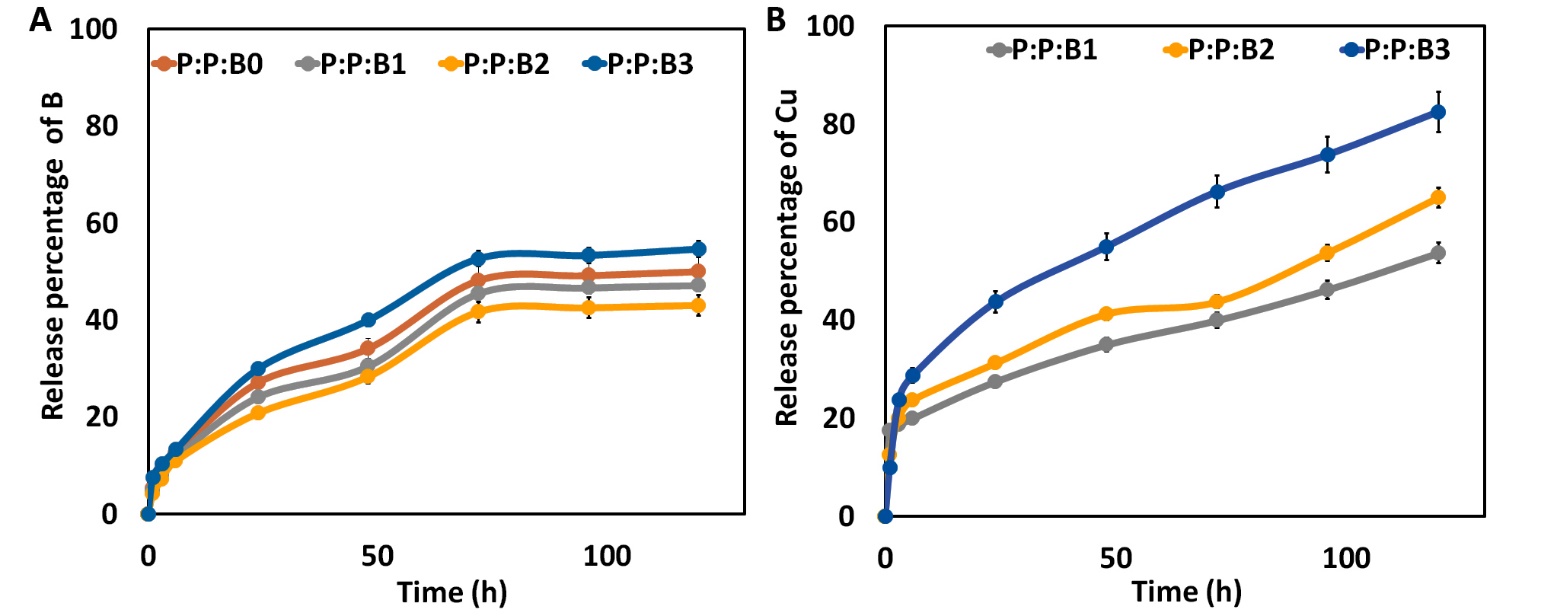


***Figure S2.*** *(A) The release percentage of B and (B) The release percentage of Cu released from the P:P:Bs grafts into the medium as a function of the immersion time of the grafts in the culture medium*


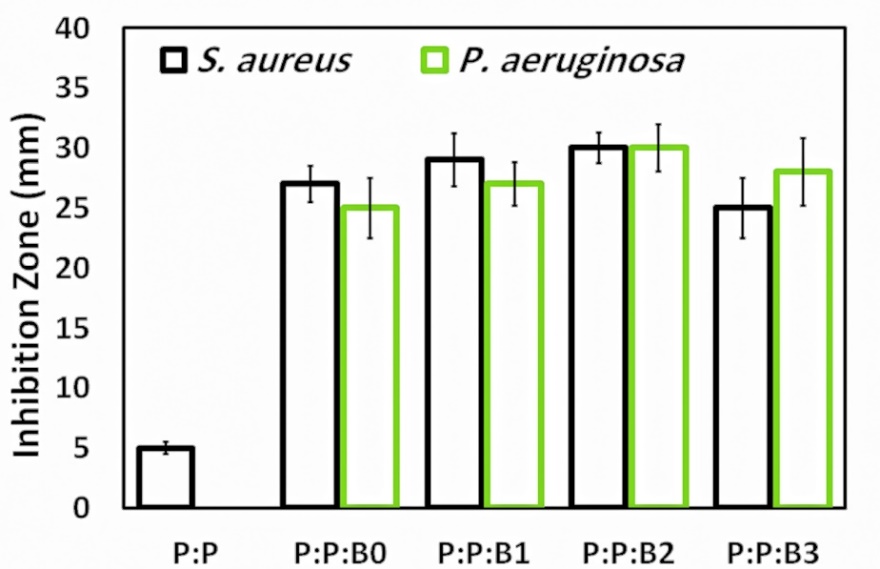


***Figure S3.*** *Diameters of bacterial growth inhibition zone.*

*Eq. (S1):*

| $\frac{\left( m_{eq}-m_{BGs} \right)-(m_{d}-m_{BGs})}{m_{d}-m_{BGs}}\times100=\frac{m_{eq}-m_{d}}{m_{d}\left( 1-{\%wtBGs} \right)}\times100$ | (S1) |
| --- | --- |

*Eq. (S2):*

| $\nu_{2}=\left[ 1+\left( \frac{m_{eq}-m_{d}}{m_{d}} \right)\left( \frac{\rho2}{\rho1} \right) \right]^{-1}$ | (S2) |
| --- | --- |

where polymer and solvents density are shown by $\rho_{1}$ and $\rho_{2}$, respectively.

*Eq. (S3):*

| $n=\frac{\ln\left( 1-\nu_{2} \right)+\nu_{2}+\chi\nu_{2}^{2}}{\nu_{1}(\frac{2\nu_{2}}{3}-\nu_{2}^{\frac{1}{3}})}$ | (S3) |
| --- | --- |

where strand density, polymer-solvent interaction parameter, and molar volume of the solvent are shown with$n$, $\chi$ (Flory-Huggins parameter) and $\nu_{1}$, respectively.

*Eq. (S4):*

| $E=3RTn$ | (S4) |
| --- | --- |

while T is the absolute temperature and $R$ is the universal gas constant.
